# Environmental memory in macrophages: a PRISMA-guided systematic review and the macrophage environmental memory systems framework

**DOI:** 10.3389/fsysb.2026.1955135

**Published:** 2026-10-21

**Authors:** Douglas M. Ruden

**Affiliations:** Department of Obstetrics and Gynecology, Charles S. Mott Center for Human Growth and Development, Institute of Environmental Health Sciences, Wayne State University, Detroit, MI, United States

**Keywords:** environmental memory, epigenetics, extracellular vesicles, immunometabolism, macrophages, MEMS, non-coding RNA, systems immunology

## Abstract

**Background:**

Macrophages respond dynamically to environmental and tissue-derived stressors, but the extent to which these responses constitute persistent environmental memory remains unclear. Studies of trained immunity, epigenetic regulation, non-coding RNAs, transposable elements, extracellular vesicles (EVs), and immunometabolism suggest multiple mechanisms that may contribute to persistent macrophage states, yet these fields have largely developed independently. We therefore systematically evaluated evidence for macrophage environmental memory and used the resulting evidence base to develop the Macrophage Environmental Memory Systems (MEMS) Framework.

**Methods:**

A PRISMA-guided systematic review of PubMed and Web of Science was completed on 1 August 2026. Searches identified 1,405 records; after removal of 404 duplicates, 1,001 unique records underwent screening. Following title/abstract screening and final evidence audit, 119 primary studies were included. Studies were classified as Direct when a prior stressor induced a macrophage or monocyte molecular or functional state that persisted after removal of the initiating stimulus, was maintained over time or through cell division, or altered the response to a subsequent challenge; Mechanistic when they demonstrated a relevant macrophage or monocyte mechanism without directly establishing persistence or recall; or Contextual when they provided broader mechanistic support. Studies were assigned to six non-mutually exclusive MEMS modules.

**Results:**

Of the 119 included studies, only 4 met the stringent criteria for Direct evidence, whereas 50 were Mechanistic and 65 Contextual, revealing a substantial gap between the extensive literature describing environmentally responsive macrophage mechanisms and evidence directly demonstrating persistent environmental memory. Module assignments overlapped and included transposable element regulation (n = 107), metabolic and signaling reprogramming (n = 86), epigenetic regulation (n = 33), systems integration (n = 17), non-coding RNA regulation (n = 14), and EV-mediated communication (n = 7). The evidence therefore identifies numerous candidate mechanisms that may contribute to the acquisition, maintenance, integration, or communication of persistent macrophage states, while demonstrating that direct evidence of persistence remains limited.

**Conclusion:**

The available literature provides broad mechanistic support for biological processes potentially relevant to macrophage environmental memory but comparatively little direct evidence of persistence or altered subsequent responses. The resulting MEMS Framework organizes this heterogeneous evidence into six interacting molecular systems and is proposed as an evidence-organizing and hypothesis-generating framework rather than a validated six-component biological pathway. Prospective exposure-withdrawal, longitudinal, lineage-tracing, perturbational, and rechallenge studies are needed to determine which mechanisms causally establish and maintain persistent macrophage states. The Macrophage-Mediated Molecular Memory (M3) hypothesis represents a downstream, experimentally testable extension of MEMS involving potential communication of environmentally acquired molecular information and remains distinct from evidence establishing the MEMS Framework itself.

## 1 Introduction

Macrophages are highly plastic innate immune cells that continuously integrate signals from their local and systemic environments. In addition to responding to infection, inflammation, tissue injury, metabolic perturbation, toxicants, and other environmental challenges, macrophages can undergo molecular and functional reprogramming that influences subsequent cellular behavior. Persistent forms of such reprogramming are increasingly recognized within the broader field of innate immune memory and may influence inflammatory responses, tissue repair, host defense, and disease susceptibility [reviewed in (Daskalaki et al., 2025)]. Unlike classical adaptive immune memory, these responses do not depend on antigen-specific lymphocyte receptors but instead involve changes in cellular regulatory states.

The concept of macrophage environmental memory overlaps with, but is broader than, trained immunity (Daskalaki et al., 2025; Ferreira et al., 2024). Trained immunity describes long-lasting functional reprogramming of innate immune cells following an initial stimulus, resulting in an altered response to subsequent stimulation. Epigenetic and metabolic reprogramming are among the principal mechanisms proposed to support these persistent states (Daskalaki et al., 2025; Ferreira et al., 2024). However, macrophages also encounter prolonged or recurrent changes in their cellular environments during infection, inflammatory disease, metabolic dysfunction, cancer, tissue injury, and exposure to environmental agents. The molecular responses associated with these conditions encompass chromatin regulation, non-coding RNAs, transposable elements, inflammatory and metabolic signaling, and intercellular communication (Neidhart et al., 2000; Geng et al., 2025; Bermudez et al., 2015; Tassetto et al., 2017; Zhong et al., 2024).

A critical distinction is therefore required between an environmental response and environmental memory. Activation, polarization, differentiation, or a molecular phenotype detected during an ongoing exposure or disease state does not by itself demonstrate memory. For the purposes of this review, environmental memory requires evidence that a molecular or functional state induced by a prior stressor persists after removal of the initiating stimulus, is maintained over time or through cell division, or alters the response to a subsequent challenge. Rechallenge provides particularly strong evidence of memory but is not required when persistence after removal of the initiating signal is directly demonstrated. This operational distinction is central to the systematic review because much of the macrophage literature describes mechanisms potentially capable of supporting persistent states without experimentally demonstrating persistence itself.

### 1.1 Molecular mechanisms potentially supporting macrophage environmental memory

Persistent macrophage states could potentially be supported by several interacting molecular mechanisms. Epigenetic regulation provides one plausible mechanism for stabilizing altered transcriptional states through DNA methylation, histone modification, chromatin accessibility, and associated regulatory processes (Ferreira et al., 2024). Experimental studies have demonstrated environmentally responsive changes in these mechanisms in macrophages and monocytes (Neidhart et al., 2000; Bouttier et al., 2016; Li et al., 2020; Du et al., 2025). However, environmentally induced epigenetic remodeling should not automatically be interpreted as epigenetic memory: persistence must be demonstrated after removal of the initiating condition or through an appropriate longitudinal or rechallenge design.

One particularly relevant example comes from a U937 cell-line model in which a CDKN2B-associated piRNA altered DNA and histone H3 methylation at the CDKN2B promoter, with the induced regulatory state reported to persist after removal of the initiating piRNA and through subsequent cell divisions (Wu et al., 2015). This provides Direct evidence that an RNA-induced epigenetic state can persist beyond the initiating signal, although evidence from primary macrophages remains needed.

Non-coding RNAs provide another potential regulatory layer (reviewed in (Chen et al., 2024)). MicroRNAs, long non-coding RNAs, piRNAs, and other regulatory RNAs can influence transcription, RNA stability, translation, chromatin regulation, signaling, and macrophage phenotype. Macrophage and monocyte studies have implicated several classes of regulatory RNA in environmentally responsive cellular states (Geng et al., 2025; de Araujo et al., 2019; Ma et al., 2022; Feng et al., 2025; Liu et al., 2026). The potential role of PIWI/piRNA pathways in persistent macrophage states remains comparatively understudied. This area is nevertheless of interest because small RNAs can interact with chromatin-regulatory mechanisms and transposable elements, creating potential links among several molecular systems considered in this review (Wu et al., 2015).

Transposable elements (TEs), including endogenous retroviruses (ERVs) and retrotransposons, constitute another potentially important component of this regulatory architecture (reviewed in (Russ and Iordanskiy, 2023)). TE expression or regulation is altered in macrophage-related experimental systems and disease contexts involving infection, inflammation, differentiation, cancer, toxicant exposure, and other cellular stresses (Feng et al., 2025; Herzner et al., 2021; Dopkins et al., 2023; Perron et al., 2005; Laderoute, 2020). Retroelement-derived RNA, DNA, or protein products can interact with innate immune and interferon-associated signaling pathways, linking genome regulation with inflammatory responses (Bermudez et al., 2015; Herzner et al., 2021; Dopkins et al., 2023; Garcia et al., 2024; Jiang et al., 2026).

Evidence of persistence within this domain is much more limited than evidence of TE responsiveness. In post-acute COVID-19, persistent monocyte-associated HERV transcriptional states have been reported beyond the acute phase of infection (Koo and Morrow, 2025; Koo and Morrow, 2026). In a distinct experimental system, *Drosophila* macrophage-like circulating immune cells converted viral RNA into reverse-transcribed viral information that supported continued antiviral small-RNA production after the original viral RNA was no longer detectable (Tassetto et al., 2017). These studies illustrate why TE- and retroelement-associated mechanisms are relevant to environmental memory while also emphasizing the need to distinguish persistent molecular states from TE activation occurring only during ongoing stress.

Macrophages can additionally transmit environmentally responsive molecular information through extracellular vesicles (EVs). EVs can carry proteins, lipids, RNAs, and other regulatory molecules capable of influencing recipient cells, and macrophage EV composition and biological activity can change with cellular state (Feng et al., 2025; Otsuki et al., 2021; Pantazi et al., 2022; Zhong et al., 2024). Tassetto et al. demonstrated both persistence of antiviral molecular information and EV-mediated transfer of antiviral small RNAs in *Drosophila* macrophage-like immune cells (Tassetto et al., 2017). However, transfer of molecular information is not necessarily transfer of memory. Direct evidence that EV transfer itself generates a persistent memory state in recipient cells remains limited.

### 1.2 Metabolic and signaling reprogramming

Macrophage environmental responses are also closely coupled to intracellular signaling and cellular metabolism. Changes in inflammatory signaling, innate immune sensing, mitochondrial function, glycolysis, lipid metabolism, and other metabolic processes can influence macrophage phenotype and transcriptional regulation (Bermudez et al., 2015; Herzner et al., 2021; Setoguchi et al., 1989; Leslie et al., 1991; McIntyre et al., 2003; Kumar et al., 2004). Metabolic intermediates and cellular energetic state can also interact with chromatin-regulatory processes, providing a potential connection between environmental sensing and epigenetic regulation (reviewed in (Ferreira et al., 2024; Han et al., 2025)).

The literature evaluated here, however, is substantially stronger for signaling and environmentally responsive reprogramming than for direct demonstration of persistent metabolic memory. Consequently, metabolic or signaling changes observed during active stimulation, polarization, infection, or disease were considered potentially relevant mechanisms but were not classified as Direct evidence unless persistence or altered subsequent responsiveness was demonstrated.

These observations suggest that candidate mechanisms relevant to macrophage environmental memory should not necessarily be considered in isolation. Epigenetic regulation can interact with TE expression [reviewed in (Zhang and Jagannath, 2025)]; regulatory RNAs can influence chromatin and signaling; retroelement products can activate innate immune sensors; metabolic and inflammatory signaling can influence transcriptional regulation; and EVs can communicate regulatory molecules between cells (Wu et al., 2015; Bermudez et al., 2015; Tassetto et al., 2017; Herzner et al., 2021; Zhong et al., 2024; Du et al., 2025). Such interactions provide the rationale for evaluating macrophage environmental memory using a systems-level framework. They do not, however, establish that these mechanisms collectively constitute a persistent memory system.

### 1.3 Distinguishing environmental stress responses from environmental memory

A central challenge in evaluating this literature is distinguishing environmental response from environmental memory. Macrophage activation, polarization, differentiation, or molecular changes observed during continuous exposure to a stimulus do not by themselves demonstrate memory. Similarly, altered RNA, chromatin, TE, metabolic, signaling, or EV profiles in macrophages or monocytes obtained during active disease demonstrate environmentally associated cellular states but do not necessarily establish that these states persist after the initiating environment changes.

For this review, environmental stress was defined from the perspective of the macrophage and therefore included both exogenous exposures and alterations of the cellular or tissue microenvironment (Kim et al., 2025). Relevant stressors included infection, inflammatory and autoimmune conditions, metabolic perturbation, cancer and the tumor microenvironment, tissue injury, environmental chemicals, and other disease-associated conditions (Daskalaki et al., 2025). This broad definition permits molecular responses to diverse macrophage environments to be evaluated within a common framework while maintaining a much more stringent definition of memory.

Accordingly, Direct evidence of macrophage environmental memory required demonstration that a stress-induced molecular or functional state persisted after removal of the initiating stimulus, was maintained over time or through cell division, or altered the response to a subsequent challenge. Rechallenge was considered strong evidence of memory but was not required when persistence after removal of the initiating signal was directly demonstrated. Studies identifying molecular mechanisms potentially capable of supporting persistence remained relevant to the review but were distinguished from studies directly demonstrating memory.

This operational definition was intentionally conservative. It prevents acute activation, ongoing inflammatory signaling, disease-associated phenotypes, tissue imprinting, polarization, differentiation, or continuous stimulation from being interpreted as environmental memory solely because they are accompanied by extensive molecular reprogramming. The distinction between response and memory therefore provides the evidentiary boundary used throughout this systematic review and is summarized together with the MEMS evidence hierarchy in Box 1.

### 1.4 Knowledge gap, study objective, and the MEMS framework

Despite rapid advances in macrophage biology, evidence potentially relevant to environmental memory is distributed across largely separate literatures examining trained immunity, epigenetic regulation, non-coding RNAs, TEs and ERVs, EV-mediated communication, metabolism and signaling, infection, cancer, and inflammatory disease (Daskalaki et al., 2025; Wu et al., 2015; Tassetto et al., 2017; Herzner et al., 2021; Koo and Morrow, 2025). Consequently, mechanisms potentially capable of generating, stabilizing, or communicating persistent macrophage states are often studied independently, and the extent to which they interact as components of environmental memory remains uncertain.

To address this gap, we conducted a PRISMA-guided systematic review of molecular mechanisms potentially contributing to macrophage and monocyte environmental memory. Rather than treating all environmentally induced macrophage changes as evidence of memory, studies were classified according to their relationship to the central question. Direct evidence required experimental evidence of persistence, temporal maintenance, or altered subsequent response; Mechanistic evidence identified macrophage or monocyte mechanisms potentially relevant to memory without directly demonstrating persistence or recall; and Contextual evidence provided broader mechanistic evidence relevant to interpretation of the framework.

Following screening and final evidence audit, 119 primary studies were included, comprising 4 Direct, 50 Mechanistic, and 65 Contextual studies. The evidence was organized into six interacting and non-mutually exclusive molecular domains: epigenetic regulation, non-coding RNA regulation, transposable element regulation, extracellular vesicle-mediated communication, metabolic and signaling reprogramming, and systems integration. These domains form the Macrophage Environmental Memory Systems (MEMS) Framework, proposed here as an evidence-organizing structure for evaluating and testing relationships among molecular mechanisms rather than as an established six-component biological pathway. The overall framework and relationships among the six modules are illustrated in Figure 1, the operational definition of environmental memory and relationship between MEMS and M3 are summarized in Box 1, and the evidence profile, representative mechanisms, and major knowledge gaps for each module are summarized in Table 1. The previously proposed M3 hypothesis is considered a downstream, experimentally testable extension of MEMS and remains speculative rather than evidence establishing the framework (Ruden, 2026).

BOX 1 **The Macrophage Environmental Memory Systems (MEMS) Framework and the Macrophage-Mediated Molecular Memory (M3) Hypothesis.**

The Macrophage Environmental Memory Systems (MEMS) Framework is proposed as a conceptual systems-biology model for organizing molecular mechanisms that may enable macrophages to acquire, maintain, integrate, and communicate information generated by environmental stressors. Environmental stress is defined from the perspective of the macrophage and includes both exogenous exposures and alterations of the cellular or tissue microenvironment, including infection, inflammation, metabolic perturbation, cancer and the tumor microenvironment, tissue injury, and other disease-associated conditions (Daskalaki et al., 2025).

A central feature of MEMS is the distinction between an environmental response and environmental memory. Acute activation, polarization, differentiation, tissue-associated phenotypes, or molecular changes occurring during continuous stimulation are not, by themselves, considered evidence of memory. Direct evidence requires demonstration that a stress-induced macrophage or monocyte molecular or functional state persists after removal of the initiating stimulus, is maintained over time or through cell division, or alters the response to a subsequent challenge. Rechallenge provides particularly strong evidence of memory but is not required when persistence after removal of the initiating signal is directly demonstrated. This stringent operational definition is important because the systematic review identified only 4 Direct studies among 119 included primary studies, compared with 50 Mechanistic and 65 Contextual studies.

The MEMS Framework organizes the evidence into six interconnected and non-mutually exclusive molecular systems:

1. Epigenetic regulation—DNA methylation, histone modifications, chromatin accessibility, and related mechanisms that can alter or stabilize transcriptional states (Neidhart et al., 2000; Wu et al., 2015; Bouttier et al., 2016; Li et al., 2020).
2. Non-coding RNA regulation—microRNAs, long non-coding RNAs, piRNAs, and other regulatory RNAs that influence gene expression, chromatin regulation, signaling, and macrophage phenotype (Wu et al., 2015; Geng et al., 2025; de Araujo et al., 2019; Ma et al., 2022; Feng et al., 2025).
3. Transposable element regulation—endogenous retroviruses, retrotransposons, and other repetitive elements that interact with epigenetic regulation, small-RNA pathways, innate immune sensing, and environmentally responsive cellular states (Bermudez et al., 2015; Tassetto et al., 2017; Herzner et al., 2021; Dopkins et al., 2023; Koo and Morrow, 2025; Koo and Morrow, 2026).
4. Extracellular vesicle-mediated communication—intercellular transfer of RNAs, proteins, lipids, metabolites, and other regulatory molecules (Feng et al., 2025; Tassetto et al., 2017; Otsuki et al., 2021; Pantazi et al., 2022; Zhong et al., 2024). EV-mediated information transfer is not considered memory transfer unless a persistent state is demonstrated in the recipient cell.
5. Metabolic and signaling reprogramming—interactions among immunometabolism, inflammatory and innate immune signaling, mitochondrial function, and other regulatory pathways that can alter macrophage phenotype and transcriptional state (Bermudez et al., 2015; Herzner et al., 2021; Setoguchi et al., 1989; Leslie et al., 1991; McIntyre et al., 2003; Kumar et al., 2004). Direct evidence specifically demonstrating persistent metabolic reprogramming remains limited.
6. Systems integration—interactions among multiple molecular layers that provide a framework for investigating how environmentally induced macrophage states may be acquired, maintained, or communicated (Koo and Morrow, 2025; Koo and Morrow, 2026; Jiang et al., 2017; Gerlevik et al., 2024; Du et al., 2024). Systems integration is an organizational component of MEMS and should not be interpreted as evidence that individual studies experimentally tested or validated the complete MEMS Framework.

These modules represent candidate interacting biological systems rather than six obligatory components of a single molecular pathway. Studies were therefore classified separately as Direct, Mechanistic, or Contextual according to how directly they addressed environmental memory. The number of studies assigned to a module should not be interpreted as equivalent to the strength of evidence that the module generates persistent memory. Because studies could contribute to multiple modules, module assignments are non-mutually exclusive. The framework and relationships among the modules are illustrated in Figure 1, and their evidence profiles are summarized in Table 1.

Within this broader framework, the previously proposed Macrophage-Mediated Molecular Memory (M3) hypothesis represents a downstream, experimentally testable hypothesis rather than evidence establishing MEMS (Ruden, 2026). M3 proposes that macrophages may acquire environmentally induced molecular information, potentially including regulatory small RNAs such as piRNAs, and communicate these signals to other cells through extracellular vesicles. Under appropriate biological conditions, such communication could potentially extend to germ cells and contribute to epigenetic inheritance (Ruden, 2026). These proposed intercellular and intergenerational mechanisms remain speculative and require direct experimental validation. MEMS does not require the M3 hypothesis to be correct; M3 represents one potential downstream consequence of the acquisition, persistence, and communication of environmentally induced molecular information organized within the MEMS Framework.

**FIGURE 1:**
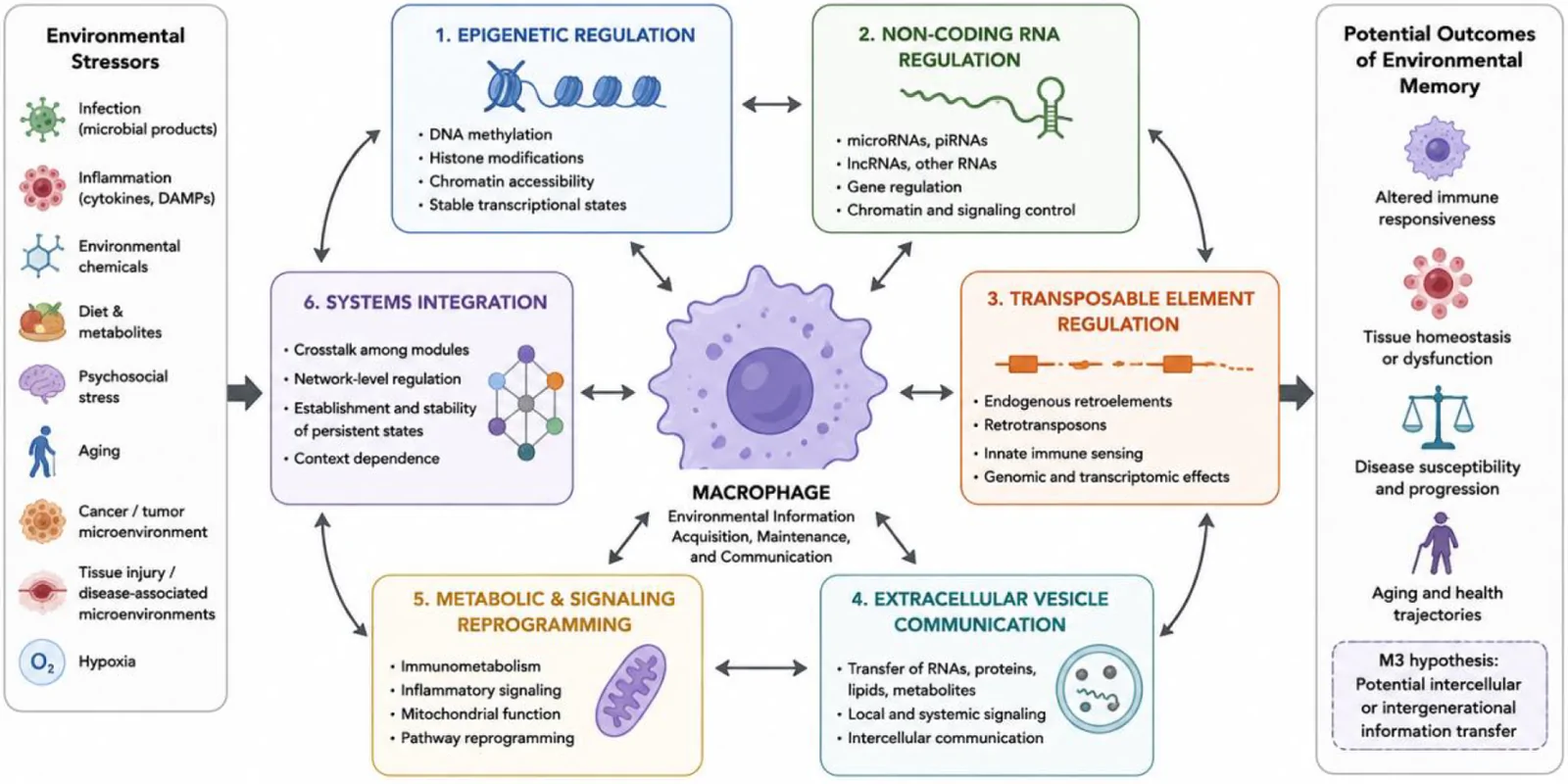
The Macrophage Environmental Memory Systems (MEMS) Framework. Environmental stressors interact with six interconnected molecular systems that may contribute to macrophage environmental memory: epigenetic regulation, non-coding RNA regulation, transposable element regulation, extracellular vesicle-mediated communication, metabolic and signaling reprogramming, and systems integration. These modules are proposed to support the acquisition, maintenance, and communication of environmentally induced molecular information and may influence immune responsiveness, tissue homeostasis or dysfunction, disease susceptibility, and aging. The MEMS Framework is a conceptual model and does not imply that all six modules have been experimentally demonstrated to generate persistent memory. The Macrophage-Mediated Molecular Memory (M3) hypothesis represents a downstream, testable extension involving potential intercellular or intergenerational transfer of environmentally acquired molecular information. Abbreviations: DAMPs, damage-associated molecular patterns; EV, extracellular vesicle; lncRNA, long non-coding RNA; MEMS, Macrophage Environmental Memory Systems; M3, Macrophage-Mediated Molecular Memory; piRNA, PIWI-interacting RNA; RNA, ribonucleic acid.

**TABLE 1:** Overview of the macrophage environmental memory systems (MEMS) framework.

MEMS module	Evidence profile	Representative mechanisms	Potential relevance to environmental memory	Major evidence gap
Epigenetic regulation	33: 2 direct; 13 mechanistic; 18 contextual	DNA methylation/demethylation; histone modifications; chromatin accessibility and architecture; epigenetic regulatory enzymes	Candidate mechanisms for maintaining environmentally induced transcriptional states after the initiating stimulus	Most studies demonstrate environmentally responsive epigenetic changes rather than persistence after stimulus withdrawal or recall
Non-coding RNA regulation	14: 1 direct; 9 mechanistic; 4 contextual	miRNAs; lncRNAs; piRNAs; RNA-mediated transcriptional and post-transcriptional regulation	Regulatory RNAs may influence chromatin, gene expression, TE activity, and signaling and could contribute to maintenance of persistent states	Only one direct study; limited evidence that ncRNA-induced states persist after removal of the initiating signal
Transposable element regulation	107: 3 direct; 41 mechanistic; 63 contextual	LINE-1; ERVs; alu elements; reverse transcription; TE-derived RNA/DNA; innate nucleic-acid sensing	TEs may connect environmental stress, chromatin regulation, ncRNAs, and innate immune signaling	Largest evidence base but predominantly mechanistic/Contextual; TE activation during ongoing disease or exposure is not itself evidence of memory
Extracellular vesicle-mediated communication	7: 1 direct; 4 mechanistic; 2 contextual	EV cargo containing ncRNAs, TE-derived RNAs, proteins, lipids, and other regulatory molecules	Potential mechanism for communicating environmentally acquired molecular information between cells	Information transfer has rarely been shown to produce a persistent state in recipient cells; information transfer ≠ memory transfer
Metabolic and signaling reprogramming	86: 4 direct; 36 mechanistic; 46 contextual	Innate immune sensing; interferon and inflammatory signaling; differentiation/polarization pathways; metabolic regulation	Signaling and metabolic networks may participate in acquisition or maintenance of environmentally induced macrophage states	Evidence is dominated by signaling rather than metabolism; direct evidence specifically demonstrating persistent metabolic reprogramming remains limited
Systems integration	17: 2 direct; 7 mechanistic; 8 contextual	Multi-omic regulation; cross-module interactions; coordinated transcriptional, epigenetic, signaling, and cellular networks	Provides an approach for testing whether persistent states involve coordinated changes across multiple MEMS modules	Systems integration is partly an organizational construct of MEMS; multi-omic association does not demonstrate a causal integrated memory pathway

The six interacting MEMS, modules summarize molecular systems that may contribute to the acquisition, maintenance, integration, or communication of environmentally induced macrophage states. The evidence profile reports the number of studies assigned to each module and their classification as Direct, Mechanistic, or Contextual evidence. Because individual studies could contribute to more than one module, module assignments are non-mutually exclusive and do not sum to the 119 included primary studies. Direct evidence required that an induced macrophage or monocyte molecular or functional state persist after removal of the initiating stimulus, be maintained over time or through cell division, or alter the response to a subsequent challenge. Mechanistic evidence demonstrated macrophage or monocyte mechanisms relevant to MEMS, without directly establishing persistence or recall, whereas Contextual evidence provided broader mechanistically relevant support for the framework. Module assignment indicates relevance to the MEMS, evidence synthesis and should not be interpreted as evidence that the module independently generates environmental memory or as an assessment of study quality. Study-level evidence and classifications are provided in Supplementary Table S2, integrated module-level synthesis in Supplementary Table S3, and citation mapping of the 119 included primary studies in Supplementary Table S4.

Abbreviations: Alu, Alu short interspersed nuclear element; DNA, deoxyribonucleic acid; ERV, endogenous retrovirus; EV, extracellular vesicle; LINE-1, long interspersed nuclear element-1; lncRNA, long non-coding RNA; MEMS, macrophage environmental memory systems; miRNA, microRNA; ncRNA, non-coding RNA; piRNA, PIWI-interacting RNA; RNA, ribonucleic acid; TE, transposable element.

## 2 Materials and methods

This systematic review was conducted in accordance with the Preferred Reporting Items for Systematic Reviews and Meta-Analyses (PRISMA) 2020 framework to evaluate molecular mechanisms relevant to macrophage environmental memory (Page et al., 2021). Because the literature spans immunology, epigenetics, RNA biology, transposable-element regulation, extracellular-vesicle signaling, metabolism, and disease-associated macrophage biology, evidence was synthesized across these traditionally separate fields and organized within the six-module Macrophage Environmental Memory Systems (MEMS) Framework.

For purposes of this review, an environmental stressor was defined from the perspective of the macrophage or monocyte and included both exogenous exposures and alterations of the cellular or tissue microenvironment. Relevant stressors therefore included infection, inflammatory and autoimmune conditions, metabolic perturbation, cancer and the tumor microenvironment, tissue injury, and environmental exposures. This definition was intended to capture the diverse environments encountered by macrophages while distinguishing exposure to a stressor from evidence that the resulting cellular state constitutes environmental memory.

### 2.1 Literature search strategy

A systematic literature search was conducted in PubMed and Web of Science, with the final searches performed on 1 August 2026, to identify studies relevant to macrophage and monocyte environmental memory and its underlying molecular mechanisms. The database-specific searches combined macrophage or monocyte terms with terms encompassing environmental memory, trained immunity, epigenetic regulation, DNA methylation, histones, chromatin, non-coding RNAs, miRNAs, piRNAs, PIWI proteins, transposable elements, retrotransposons, extracellular vesicles, exosomes, and metabolism. The exact database-specific search strings and search date are provided in Supplementary Table S1.

The PubMed search identified 738 records, and the Web of Science search identified 667 records, yielding 1,405 records before deduplication. Search results were imported into EndNote 21 (Clarivate) for reference management and duplicate removal. Removal of 404 duplicate records yielded 1,001 unique records for title and abstract screening. The final systematic-review evidence set therefore comprised 119 primary studies.

The complete search strategy and study-selection audit trail are provided in Supplementary Table S1, study-level inclusion decisions and evidence classifications in Supplementary Table S2, and the overall study-selection process in Figure 2.

**FIGURE 2:**
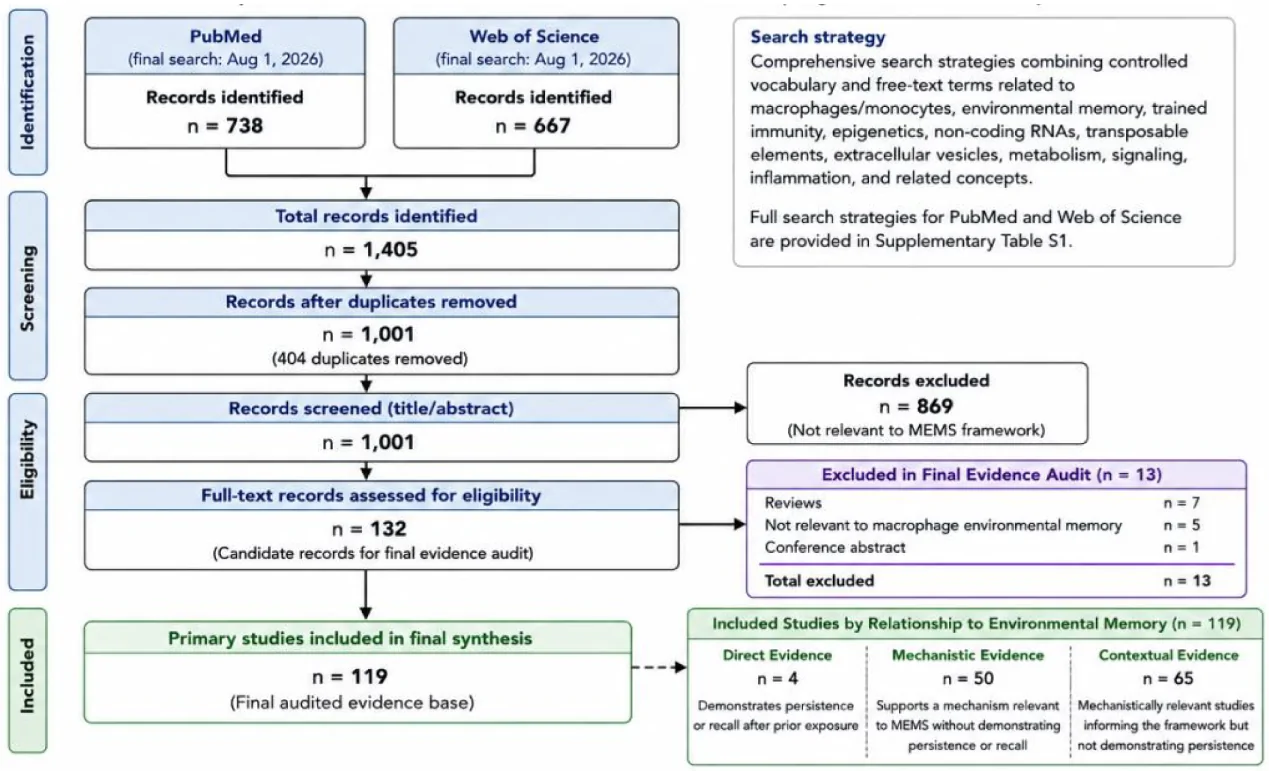
PRISMA flow diagram of study identification, screening, and final evidence audit. Systematic searches of PubMed and Web of Science, with final searches performed on 1 August 2026, identified 1,405 records (738 and 667, respectively). After removal of 404 duplicates, 1,001 unique records underwent title and abstract screening, of which 882 were excluded. The final qualitative synthesis therefore included 119 primary studies. Studies were classified according to their relationship to macrophage environmental memory as Direct (n = 4), Mechanistic (n = 50), or Contextual (n = 65). Detailed database search strategies and the study-selection audit trail are provided in Supplementary Table S1, with study-level classifications provided in Supplementary Table S2. Abbreviations: MEMS, Macrophage Environmental Memory Systems; PRISMA, Preferred Reporting Items for Systematic Reviews and Meta-Analyses.

### 2.2 Eligibility criteria

Studies were eligible for inclusion if they provided primary experimental or observational evidence relevant to macrophages, monocytes, or closely related monocyte-derived cells and investigated molecular or cellular mechanisms relevant to macrophage environmental memory or to one or more components of the Macrophage Environmental Memory Systems (MEMS) Framework. Eligible experimental systems included human samples, animal models, primary cells, cell lines, and other relevant biological models.

Environmental stress was defined from the perspective of the macrophage and included both exogenous exposures and alterations of the cellular or tissue microenvironment. Relevant stressors therefore included microbial infection, inflammation and autoimmune disease, metabolic perturbation, cancer and the tumor microenvironment, tissue injury, environmental chemicals, and other disease-associated conditions. This broad definition was used because macrophages encounter and respond to changes in their immediate microenvironment regardless of whether the initiating perturbation originates externally or within the organism (Daskalaki et al., 2025). Importantly, broad inclusion of environmental stressors did not broaden the operational definition of environmental memory.

Eligible molecular mechanisms included epigenetic regulation, DNA methylation, histone modification and chromatin remodeling; non-coding RNA regulation, including PIWI proteins and PIWI-interacting RNAs (piRNAs); transposable elements and endogenous retroviruses; extracellular vesicle (EV)-mediated communication; metabolic and signaling reprogramming; and systems-level interactions among these processes. Studies were not required to demonstrate environmental memory directly because a principal objective of the review was to evaluate both direct evidence of persistent memory and the broader mechanistic literature potentially capable of supporting such states.

To distinguish environmental responses from environmental memory, included studies were assigned to one of three evidence-relationship categories. Direct evidence required experimental evidence that a prior stressor induced a macrophage or monocyte molecular or functional state that persisted after removal of the initiating stimulus, was maintained over time or through cell division, or altered the response to a subsequent challenge. Rechallenge was considered particularly strong evidence of memory but was not required when persistence after removal of the initiating signal was directly demonstrated. Mechanistic evidence comprised macrophage or monocyte studies demonstrating a molecular or cellular mechanism relevant to MEMS without directly demonstrating persistence or recall. Contextual evidence comprised mechanistically relevant studies that informed interpretation of the framework but did not themselves constitute direct evidence of macrophage environmental memory.

Accordingly, macrophage activation, polarization, differentiation, trained or tolerant phenotypes, or molecular alterations observed only during continuing stimulation were not classified as Direct evidence in the absence of demonstrated persistence, temporal maintenance, or altered subsequent response. Similarly, disease-associated macrophage states were not interpreted as environmental memory solely because they differed from reference or control populations. These distinctions were applied specifically to avoid conflating acute activation, ongoing adaptation, tissue imprinting, or continuous stimulation with persistent environmental memory.

Primary research articles were eligible for the final evidence synthesis. Reviews and conceptual articles could be used as background sources but were excluded from the primary evidence set. Conference abstracts without an eligible full publication, editorials, commentaries, duplicate reports, studies lacking sufficient relevance to macrophage or monocyte biology, and studies lacking sufficient mechanistic relevance to the MEMS Framework were excluded. The final primary evidence set comprised 119 studies. The 13 candidate records excluded during the final evidence audit were retained in Supplementary Table S2 together with their reasons for exclusion to provide a transparent audit trail.

### 2.3 Data extraction and annotation

Data from each included study were entered into a standardized evidence atlas developed for this review. Extracted information included bibliographic information, experimental model and evidence level, environmental exposure or biological stressor, macrophage or monocyte phenotype, molecular mechanisms investigated, principal findings, and relevance to macrophage environmental memory.

Each study was annotated for its contribution to one or more of the six MEMS modules: (i) epigenetic regulation, (ii) non-coding RNA regulation, (iii) transposable element regulation, (iv) extracellular vesicle-mediated communication, (v) metabolic and signaling reprogramming, and (vi) systems integration. Because these modules represent interacting biological processes rather than mutually exclusive categories, individual studies could be assigned to more than one module.

Attention was given to evidence connecting different MEMS modules, including interactions between epigenetic regulation and transposable elements, ncRNAs and chromatin regulation, transposable elements and innate immune sensing, metabolic or inflammatory signaling and epigenetic regulation, and EV-mediated transfer of regulatory molecules (Wu et al., 2015; Tassetto et al., 2017; Herzner et al., 2021; Zhong et al., 2024; Du et al., 2025). These cross-module relationships were annotated to distinguish studies investigating individual mechanisms from those providing evidence relevant to systems-level integration. Cross-module assignment did not imply that a study had experimentally demonstrated the MEMS Framework or a persistent environmental-memory state.

The final evidence atlas contains 119 primary studies, and study-level data, module assignments, and evidence classifications are provided in Supplementary Table S2. The 13 excluded candidate records are also retained in Supplementary Table S2 to document the final eligibility audit.

### 2.4 Evidence classification and synthesis

Evidence was synthesized according to molecular systems rather than disease category or experimental methodology. This approach permitted findings obtained across diverse environmental stressors, diseases, experimental models, and biological contexts to be evaluated within common mechanistic domains while preserving differences in the directness of their relationship to environmental memory.

The 119 included studies were stratified according to their relationship to macrophage environmental memory rather than according to methodological quality. Direct evidence (n = 4) demonstrated persistence after removal of an initiating stressor, maintenance over time or through cell division, or an altered response to a subsequent challenge. Mechanistic evidence (n = 50) demonstrated macrophage or monocyte mechanisms relevant to MEMS without directly establishing persistence or recall. Contextual evidence (n = 65) provided broader mechanistically relevant evidence informing the framework without directly demonstrating macrophage environmental memory.

Module assignments were summarized quantitatively. Among the 119 included studies, 33 contributed to epigenetic regulation, 14 to non-coding RNA regulation, 107 to transposable element regulation, 7 to extracellular vesicle-mediated communication, 86 to metabolic and signaling reprogramming, and 17 to systems integration. Because individual studies could contribute to multiple MEMS modules, these counts are non-mutually exclusive and therefore do not sum to 119.

Evidence relationships within the individual modules were also summarized. Epigenetic regulation comprised 2 Direct, 13 Mechanistic, and 18 Contextual studies; non-coding RNA regulation, 1 Direct, 9 Mechanistic, and 4 Contextual; transposable element regulation, 3 Direct, 41 Mechanistic, and 63 Contextual; extracellular vesicle-mediated communication, 1 Direct, 4 Mechanistic, and 2 Contextual; metabolic and signaling reprogramming, 4 Direct, 36 Mechanistic, and 46 Contextual; and systems integration, 2 Direct, 7 Mechanistic, and 8 Contextual. Because of overlapping module assignments, a Direct study could contribute to more than one module. These distributions are summarized in Table 1 and Supplementary Table S3.

Evidence was synthesized qualitatively rather than by meta-analysis because of substantial heterogeneity in environmental stressors, disease contexts, biological models, macrophage and monocyte populations, experimental designs, exposure durations, follow-up periods, and molecular endpoints. The Direct, Mechanistic, and Contextual categories were therefore used to describe how directly each study addressed environmental memory, rather than as a formal numerical grading system for methodological quality.

### 2.5 Development of the MEMS conceptual framework

The MEMS Framework was developed through iterative synthesis of the evidence identified in the systematic review rather than being specified in advance as a validated biological pathway. Recurring relationships were identified among epigenetic regulation, non-coding RNA regulation, transposable element regulation, extracellular vesicle-mediated communication, metabolic and signaling reprogramming, and systems integration. These recurring mechanistic relationships formed the basis of the six-module framework.

The six modules should not be interpreted as independent, sequential, or obligatory components of a single molecular pathway. Instead, MEMS provides a systems-level organizational structure for evaluating mechanisms that may participate individually or collectively in the acquisition, maintenance, or communication of environmentally induced macrophage states. The framework therefore accommodates both established biological mechanisms and emerging mechanistic relationships while retaining explicit distinctions in the strength and directness of their relationship to.

The framework also distinguishes candidate mechanisms from demonstrated causal relationships. For example, altered EV cargo demonstrates intercellular information transfer but does not establish transfer of persistent memory unless a durable recipient-cell state is demonstrated. Similarly, transposable-element expression, epigenetic remodeling, macrophage polarization, metabolic adaptation, or multi-omic differences during an ongoing disease or exposure may identify mechanisms relevant to MEMS but do not themselves demonstrate memory. The previously proposed M3 hypothesis was treated as a downstream, testable extension of MEMS rather than as evidence used to establish the framework (Ruden, 2026).

### 2.6 Reporting, evidence quality, and methodological limitations

This systematic review was reported according to the PRISMA 2020 framework (Page et al., 2021). Database-specific search strategies, including the final search date of 1 August 2026, and the study-selection audit trail are provided in Supplementary Table S1. Study-level eligibility decisions, evidence annotations, and MEMS-module assignments are provided in Supplementary Table S2, the integrated module-level evidence synthesis is provided in Supplementary Table S3, and the mapping of all 119 included primary studies to their primary and secondary citation locations in the Results synthesis is provided in Supplementary Table S4. The overall study-selection process is summarized in Figure 2.

The evidence base encompassed heterogeneous human observational studies, animal experiments, primary-cell studies, cell-line experiments, and other mechanistic investigations. Because these study designs require different approaches to methodological appraisal, a single conventional risk-of-bias instrument was not applied uniformly across the entire evidence set. The Direct, Mechanistic, and Contextual classifications should therefore not be interpreted as formal assessments of methodological quality or risk of bias. Rather, they describe how directly each study addresses the central question of macrophage environmental memory. Similarly, assignment to a MEMS module reflects biological and mechanistic relevance rather than study quality.

Several methodological limitations should be considered when interpreting the findings. First, study identification, screening, eligibility assessment, data extraction, annotation, and final evidence classification were conducted by a single author rather than independently by two reviewers, creating the possibility of selection or classification bias. The addition of Supplementary Table S4 further provides a study-level audit trail linking each included primary study to its location in the narrative Results synthesis. The final evidence set comprised 4 Direct, 50 Mechanistic, and 65 Contextual studies.

Second, the evidence base was substantially heterogeneous. Studies differed in environmental stressors, disease contexts, macrophage or monocyte populations, experimental models, exposure durations, molecular endpoints, and follow-up periods. This heterogeneity precluded meaningful quantitative meta-analysis and limited direct comparison across studies. Consequently, synthesis was qualitative and focused on recurring molecular mechanisms, evidence relationships, and interactions among the six MEMS modules.

Third, most included studies were not specifically designed to test environmental memory. Many investigated molecular mechanisms during infection, inflammation, cancer, metabolic disease, tissue injury, or other altered microenvironments and therefore provided Mechanistic or Contextual rather than Direct evidence. The small Direct evidence set (n = 4) is consequently both a limitation and an important finding of the systematic review: despite a large literature describing environmentally responsive macrophage mechanisms, relatively few studies directly tested whether induced states persisted after withdrawal of the initiating stimulus, were maintained over time or through cell division, or modified responses to subsequent challenges.

Finally, because MEMS was developed through synthesis of a heterogeneous literature rather than prospectively specified as a validated biological model, relationships among its six modules should be interpreted as an evidence-organizing framework and source of experimentally testable hypotheses, not as proof of a unified six-component pathway. Conclusions concerning persistent macrophage environmental memory were therefore restricted primarily to findings supported by Direct evidence. Mechanistic and Contextual studies were used to evaluate biological plausibility, identify candidate mechanisms and cross-module relationships, and generate experimentally testable predictions. This evidence hierarchy was maintained to avoid extending observations of acute activation, polarization, continuous stimulation, disease-associated states, EV-mediated information transfer, or systems-level association into claims of persistent environmental memory without direct experimental support.

## 3 Results

The systematic search identified 1,405 records, of which 1,001 remained after removal of 404 duplicates and underwent title and abstract screening. Following screening and the final evidence audit, 119 primary studies met the criteria for inclusion in the qualitative synthesis (Figure 2; Supplementary Tables S1, S2). The included literature encompassed diverse environmental and biological contexts, including infection, inflammation, metabolic perturbation, cancer and the tumor microenvironment, tissue injury, aging, and other disease-associated alterations of the macrophage microenvironment. The studies likewise employed heterogeneous human, animal, primary-cell, and cell-line systems and examined molecular processes ranging from chromatin and non-coding RNA regulation to transposable elements, innate immune signaling, metabolism, and extracellular-vesicle communication. Of the 119 included studies, only 4 met the criteria for Direct evidence, whereas 50 were Mechanistic and 65 Contextual. Thus, most of the literature identifies molecular mechanisms potentially relevant to persistent macrophage states without directly demonstrating environmental memory.

The Results are organized first by the overall characteristics and strength of the evidence base and then according to the six MEMS modules. Because individual studies could contribute to multiple modules, module-specific counts overlap and should not be summed to derive the 119-study total. Study-level classifications and module assignments are provided in Supplementary Table S2, the integrated module-level synthesis in Supplementary Table S3, and the mapping of all 119 included primary studies to their primary and secondary citation locations in the Results in Supplementary Table S4.

### 3.1 Characteristics and strength of the evidence base

The 119 included studies constituted a heterogeneous evidence base spanning human observational studies, animal models, primary macrophages and monocytes, and cell-based experimental systems. Human studies examined macrophage- or monocyte-associated molecular states across infection, inflammatory and autoimmune disease, metabolic disorders, cancer, aging, and other altered tissue environments. Experimental animal and cellular studies provided complementary mechanistic evidence by permitting more direct interrogation of chromatin regulation, ncRNAs, TEs, innate immune signaling, metabolism, and EV-mediated communication.

The distribution of studies among MEMS modules was highly uneven. Transposable element regulation represented the largest module (107 studies), followed by metabolic and signaling reprogramming (86), epigenetic regulation (33), systems integration (17), non-coding RNA regulation (14), and extracellular vesicle-mediated communication (7) (Table 1; Supplementary Table S3). Because individual studies frequently investigated mechanisms relevant to multiple domains, these values represent overlapping assignments rather than mutually exclusive study categories. The large TE and metabolic/signaling literatures therefore indicate substantial mechanistic evidence relevant to these systems but should not be interpreted as evidence that either system independently generates persistent environmental memory. The final module counts and evidence distributions are consistent with the revised Methods.

#### 3.1.1 Direct evidence of macrophage environmental memory

Only four studies satisfied the operational criteria for Direct evidence. These studies are particularly informative because each demonstrated persistence or maintenance of environmentally induced molecular information beyond the immediate initiating signal rather than simply characterizing macrophages or monocytes during active stimulation or disease.

In a human U937 cell-line model, a CDKN2B-associated piRNA altered DNA and histone H3 methylation at the CDKN2B promoter, and the induced regulatory state was reported to persist after removal of the initiating piRNA and through subsequent cell divisions (Wu et al., 2015). This study therefore provides Direct evidence that an ncRNA-initiated epigenetic state can outlast the initiating molecular signal. Because the experiment used U937 cells, however, it should be interpreted as cell-line evidence rather than direct demonstration in primary human macrophages.

A second Direct study used *Drosophila* macrophage-like circulating immune cells and demonstrated conversion of viral RNA into reverse-transcribed, via endogenous transposon reverse transcriptase, viral information that supported continued antiviral small-RNA production after the original viral RNA was no longer detectable (Tassetto et al., 2017). The study additionally demonstrated EV-mediated transfer of antiviral small RNAs, linking reverse-transcription/TE-related processes, small-RNA biology, and intercellular communication (Tassetto et al., 2017). It therefore provides Direct evidence of persistence of environmentally acquired antiviral molecular information together with EV-mediated communication, although it does not establish that EV transfer itself generates a persistent memory state in recipient cells.

The remaining two Direct studies examined persistent molecular states following SARS-CoV-2 infection. One identified persistent amplification of selected autonomous HERV loci in monocytes from individuals with post-acute sequelae of COVID-19, accompanied by altered transcription in surrounding host genomic regions (Koo and Morrow, 2025). A subsequent study extended these observations to a persistent HERV-associated state involving a CD14^+^-xCR1^+^ mononuclear-cell phenotype and related mature dendritic-cell populations (Koo and Morrow, 2026). These studies provide Direct evidence of persistent post-acute HERV-associated transcriptional states, although the underlying mechanisms responsible for their persistence remain to be established.

Taken together, the four Direct studies demonstrate that persistent environmentally induced molecular states can occur in macrophage- or monocyte-related systems through substantially different biological contexts and mechanisms (Wu et al., 2015; Tassetto et al., 2017; Koo and Morrow, 2025; Koo and Morrow, 2026). They should not, however, be interpreted as four demonstrations of a single conserved macrophage-memory pathway. Rather, their common feature is the temporal evidence of persistence beyond the initiating signal, which distinguishes them from the much larger Mechanistic and Contextual evidence base.

#### 3.1.2 Mechanistic and Contextual evidence

The remaining 115 studies consisted of 50 Mechanistic and 65 Contextual studies. Mechanistic studies directly investigated macrophage or monocyte processes relevant to MEMS but did not demonstrate persistence or recall. These studies identified candidate mechanisms involving epigenetic regulation, ncRNAs, TEs, EV-mediated communication, innate immune and inflammatory signaling, metabolism, and interactions among these systems. Representative examples include regulation of retroelements by epigenetic mechanisms, retroelement-derived activation of innate immune pathways, environmentally responsive ncRNA networks, and EV-mediated transfer of regulatory molecules (Bouttier et al., 2016; Ma et al., 2022; Bermudez et al., 2015; Herzner et al., 2021; Pantazi et al., 2022; Zhong et al., 2024; Du et al., 2025).

Contextual studies provided broader biological evidence relevant to the MEMS Framework but did not directly establish macrophage environmental memory. This category included studies of disease-associated macrophage or monocyte states, TE and ERV expression, differentiation and polarization, signaling responses, and other environmentally responsive molecular phenotypes. These studies are useful for identifying candidate mechanisms and biological contexts in which persistent states could arise, but observations made during active disease, continuous exposure, differentiation, or ongoing stimulation were not considered evidence of memory in the absence of demonstrated persistence or recall.

The resulting evidence hierarchy therefore reveals a substantial gap between mechanistic plausibility and direct demonstration of macrophage environmental memory. The six module-specific sections that follow evaluate this distinction separately for each component of the MEMS Framework.

### 3.2 Epigenetic regulation

Epigenetic regulation was represented in 33 of the 119 included studies, comprising 2 Direct, 13 Mechanistic, and 18 Contextual studies. This module included DNA methylation and demethylation, histone modifications, chromatin accessibility and architecture, and interactions between chromatin regulation and other MEMS modules. The predominance of Mechanistic and Contextual evidence indicates that environmentally responsive epigenetic remodeling is well represented in the literature, whereas direct demonstration that such remodeling constitutes persistent macrophage environmental memory remains limited.

DNA methylation and demethylation represented a major component of the epigenetic evidence base. Studies across inflammatory, infectious, neoplastic, aging, and other disease-associated environments demonstrated changes in DNA methylation or methylation-associated regulatory states in macrophages, monocytes, or closely related myeloid populations (Greissel et al., 2015; Conti et al., 2016; Grothaus et al., 2016; Mavragani et al., 2016; Mishra et al., 2025; Zhao et al., 2017; Benayoun et al., 2019; Gualdrini et al., 2022). Collectively, these studies support the capacity of environmental and disease-associated conditions to modify epigenetic regulation, but changes measured during an ongoing exposure or disease state were classified as Mechanistic or Contextual rather than Direct evidence unless persistence was demonstrated after removal of the initiating condition.

Histone modification and chromatin organization provided a second major layer of evidence. Alterations in histone marks, nucleosome organization, regulatory-element accessibility, and higher-order chromatin structure were associated with macrophage or monocyte state transitions and environmentally responsive transcriptional programs (Olin et al., 2014; Hurst et al., 2016; Kelly et al., 2018; Fei et al., 2026). These observations are biologically compatible with mechanisms capable of stabilizing transcriptional states, but the presence of a chromatin alteration alone does not establish environmental memory. In particular, macrophage differentiation, polarization, or chromatin remodeling occurring during continued stimulation was not considered Direct evidence under the operational definition used in this review.

Epigenetic regulation also intersected extensively with transposable-element biology. DNA methylation, histone modifications, and chromatin-associated mechanisms regulate endogenous retroviruses and other repetitive elements, while environmental or disease-associated perturbations can alter this repression (Feng et al., 2025; Garcia et al., 2024; Gonzalez-Hernandez et al., 2012; Bogdan et al., 2020; Schaller et al., 2020; Chen et al., 2023; Zezulin et al., 2023; Gu et al., 2026). This cross-module relationship is important because derepressed transposable elements can generate nucleic-acid intermediates capable of activating innate immune pathways, thereby connecting chromatin regulation with inflammatory signaling. Other studies linked epigenetic regulatory states to disease-associated macrophage phenotypes and broader systems-level changes (Goffeney et al., 2025).

Only a small subset of the epigenetic literature met the stringent criteria for Direct evidence. Wu et al. demonstrated a piRNA-associated epigenetic regulatory state that persisted after removal of the initiating piRNA and through subsequent cell divisions, providing direct evidence that a transient regulatory signal can generate a maintained epigenetic state (Wu et al., 2015). Persistent epigenetic/HERV-associated alterations in post-acute monocytes provided a second line of Direct evidence within this module (Koo and Morrow, 2025). These findings differ fundamentally from studies measuring epigenetic remodeling during continuous stimulation because persistence itself was experimentally or temporally demonstrated.

Thus, the epigenetic module provides substantial evidence for environmentally responsive chromatin regulation but comparatively little Direct evidence that these alterations persist independently of the initiating stressor. Future studies should explicitly incorporate exposure withdrawal, longitudinal follow-up, cell-division tracking, and functional rechallenge together with targeted perturbation of candidate epigenetic regulators. Such designs will be necessary to distinguish transient epigenetic responses from epigenetic mechanisms that causally maintain macrophage environmental memory (Figure 3).

**FIGURE 3:**
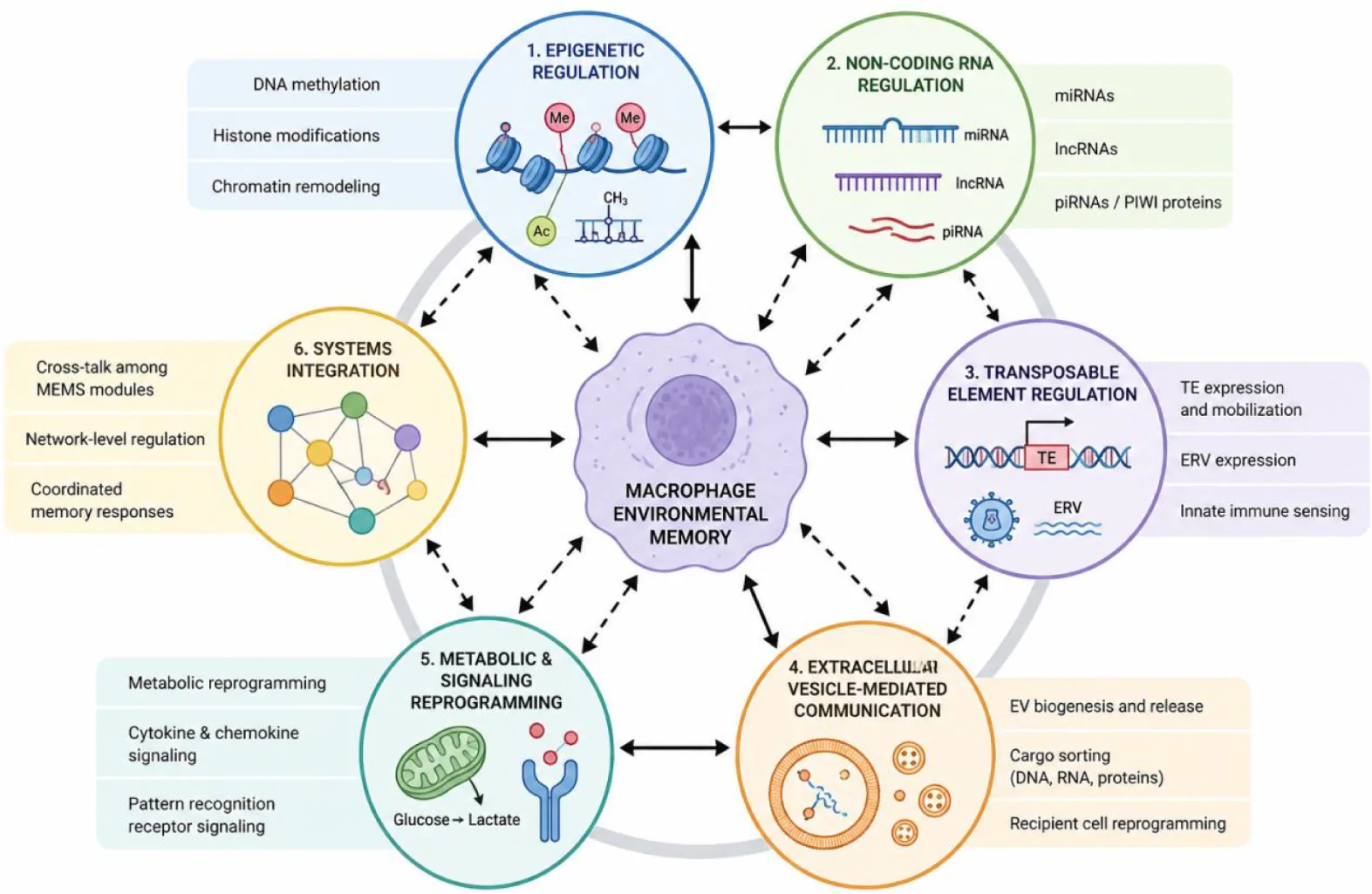
Epigenetic mechanisms potentially contributing to macrophage environmental memory. Environmental stressors can alter macrophage DNA methylation, histone modifications, chromatin accessibility and architecture, and the activity of epigenetic regulatory enzymes. Most such changes represent environmentally responsive or mechanistically relevant states rather than direct evidence of memory. Within the MEMS epigenetic module, 33 studies were identified, including 2 Direct, 13 Mechanistic, and 18 Contextual studies. Under the operational definition used in this review, an epigenetically altered state constitutes Direct evidence of environmental memory only when it persists after removal of the initiating stimulus, is maintained over time or through cell division, or alters the response to a subsequent challenge. Epigenetic regulation may interact with non-coding RNAs, transposable elements, extracellular vesicle-mediated communication, and metabolic/signaling pathways and may contribute to altered immune responsiveness, tissue function, and disease susceptibility. Abbreviations: Ac, acetylation; DNMT, DNA methyltransferase; ERV, endogenous retrovirus; EV, extracellular vesicle; HAT, histone acetyltransferase; HDAC, histone deacetylase; HMT, histone methyltransferase; KDM, histone lysine demethylase; Me, methylation; MEMS, Macrophage Environmental Memory Systems; miRNA, microRNA; ncRNA, non-coding RNA; piRNA, PIWI-interacting RNA; TE, transposable element; TET, ten-eleven translocation dioxygenase.

### 3.3 Non-coding RNA regulation

Non-coding RNA regulation was represented in 14 studies, including 1 Direct, 9 Mechanistic, and 4 Contextual studies. The evidence encompassed PIWI-interacting RNAs (piRNAs), microRNAs and other regulatory RNAs capable of influencing transcription, chromatin state, transposable-element regulation, inflammatory signaling, and macrophage phenotype. Although this literature provides substantial mechanistic support for ncRNAs as regulators of environmentally responsive macrophage states, only one study directly demonstrated persistence sufficient to satisfy the operational definition of environmental memory.

The PIWI/piRNA pathway represented a particularly important intersection between ncRNA regulation and other MEMS modules. Multiple studies identified PIWI proteins, piRNAs, or piRNA-associated mechanisms in macrophage or monocyte-related systems and linked them to gene regulation, chromatin state, inflammatory phenotype, or responses to environmental and disease-associated conditions (Geng et al., 2025; de Araujo et al., 2019; Ma et al., 2022; Feng et al., 2025; Liu et al., 2026; Kondratov et al., 2024; Allgood et al., 2025). These studies provide Mechanistic or Contextual evidence that the PIWI/piRNA system is biologically active outside its classical germline context and may contribute to macrophage regulatory plasticity. However, demonstration of PIWI or piRNA expression, or an effect on macrophage phenotype, was not considered evidence of memory unless the induced state persisted after withdrawal of the initiating signal.

The Direct evidence within this module was provided by the demonstration that a piRNA-induced regulatory state could persist after removal of the initiating piRNA and through subsequent cell divisions (Wu et al., 2015). This observation is particularly relevant to MEMS because it links a transient ncRNA signal with a maintained downstream state rather than simply demonstrating contemporaneous ncRNA regulation. It also illustrates the close mechanistic relationship between the ncRNA and epigenetic modules.

Other ncRNAs were associated with macrophage differentiation, inflammatory regulation, or environmentally responsive transcriptional programs (Wang et al., 2025). Additional studies demonstrated relationships among regulatory RNAs, extracellular-vesicle cargo, and recipient-cell responses (Feng et al., 2025; Pantazi et al., 2022; Zhong et al., 2024). These observations broaden the potential role of ncRNAs from intracellular regulators to candidate carriers of environmentally responsive molecular information. Nevertheless, altered ncRNA abundance or transfer of an ncRNA to another cell does not by itself demonstrate transfer of environmental memory.

Overall, the ncRNA evidence supports a regulatory role for small and long non-coding RNAs in macrophage environmental responses and provides plausible mechanisms connecting chromatin, transposable elements, signaling, and extracellular-vesicle communication. The major evidence gap is persistence. Prospective studies should perturb candidate ncRNAs or PIWI/piRNA pathways and then determine whether downstream molecular or functional states survive stimulus withdrawal, are maintained through cell division, or alter subsequent responses. This distinction is essential for determining which ncRNA pathways participate in environmental memory rather than transient environmental responsiveness.

### 3.4 Transposable element regulation

Transposable-element regulation was the largest MEMS module, with 107 of the 119 included studies, comprising 3 Direct, 41 Mechanistic, and 63 Contextual studies. This literature encompassed endogenous retroviruses (ERVs), human endogenous retroviruses (HERVs), porcine endogenous retroviruses (PERVs), LINEs, Alu/SINE elements, IAP elements, and other retroelements (reviewed in (Kassiotis and Stoye, 2016)). The numerical dominance of this module reflects the extensive literature linking retroelement biology to infection, inflammation, cancer, immune regulation, and disease-associated myeloid states; it should not be interpreted as evidence that transposable elements have themselves been established as a dominant mechanism of macrophage environmental memory.

One recurring theme was the responsiveness of endogenous retroviral elements to infection and inflammatory or disease-associated environments. HERV/ERV expression and regulation were altered across diverse immune and disease contexts (Feng et al., 2025; Perron et al., 2005; Laderoute, 2020; Brudek et al., 2009; Alcantara et al., 2004; Cho et al., 2004; Turcanova et al., 2009; Lask et al., 2012; Perron et al., 2012; Arru et al., 2014; Fei et al., 2014; Marttila et al., 2017; Mommert et al., 2018; Garcia-Montojo et al., 2020; Kitsou et al., 2021; Temerozo et al., 2022; Mantovani et al., 2023). These studies establish that endogenous retroelements are dynamic components of immune-cell biology, but expression during active disease or stimulation was classified as Mechanistic or Contextual unless persistence beyond the initiating condition was demonstrated.

A second group of studies addressed LINE, Alu, SINE, IAP, and other transposable-element families and their regulation in immune or environmentally responsive systems (Duhrsen et al., 1990; Sugita et al., 1990; Buchmann et al., 2007; Mommert et al., 2020). Additional work examining broader retroelement biology provided evidence that these genomic elements can respond dynamically to cellular state and environmental conditions (Gonda et al., 1987; Safi et al., 2004; Darby et al., 2011; Lipplaa et al., 2023; de Azevedo et al., 2024). Studies of PERV, a porcine ERV activated in xenotransplants, and related retroviral systems further demonstrated interactions between myeloid cells and endogenous retroviral elements (Martin et al., 2000; Specke et al., 2001; Moza et al., 2001; Leyh et al., 2003).

Transposable elements also intersected extensively with innate immune signaling. Retroelement-derived RNA or DNA can provide ligands for nucleic-acid sensing pathways, thereby linking altered genomic regulation with interferon and inflammatory responses (Bermudez et al., 2015; Herzner et al., 2021; Dopkins et al., 2023; Setoguchi et al., 1989; Leslie et al., 1991; McIntyre et al., 2003; Kumar et al., 2004; Al-Shehabi and Bannert, 2023). Epigenetic repression provides another important cross-module connection because DNA methylation, histone modifications, and chromatin organization normally restrict retroelement activity. Loss or alteration of these regulatory mechanisms can therefore simultaneously modify TE expression and inflammatory signaling (Feng et al., 2025; Garcia et al., 2024; Zezulin et al., 2023; Gu et al., 2026).

Three studies within the transposable-element module met the Direct evidence criteria. Tassetto et al. demonstrated that *Drosophila* macrophage-like circulating immune cells generated reverse-transcribed viral information and continued producing antiviral small RNAs after the original viral RNA was no longer detectable, providing evidence of persistence coupled to an antiviral function (Tassetto et al., 2017). Two post-acute studies identified persistent HERV-associated molecular states in human monocyte/myeloid populations following COVID-19 (Koo and Morrow, 2025; Koo and Morrow, 2026). These studies are qualitatively different from the much larger literature demonstrating TE expression during ongoing infection, inflammation, cancer, or other disease because they provide evidence of temporal persistence beyond the acute initiating condition.

The audit therefore substantially narrows interpretation of this large literature. Transposable elements are strongly represented as environmentally responsive regulatory and inflammatory components, but only a small fraction of the evidence directly demonstrates persistent macrophage environmental memory. Future studies should combine locus-resolved TE profiling with exposure withdrawal and rechallenge, while experimentally perturbing specific retroelements, epigenetic regulators, and nucleic-acid sensors. Such experiments would distinguish TE activation that accompanies an ongoing environmental response from TE-dependent mechanisms that causally establish or maintain environmental memory.

### 3.5 Extracellular vesicle-mediated communication

Extracellular vesicle-mediated communication was the smallest MEMS module, comprising 7 studies: 1 Direct, 4 Mechanistic, and 2 Contextual. Despite the limited number of studies, this module is conceptually important because extracellular vesicles (EVs) provide a mechanism by which environmentally responsive molecular information can be transferred between cells. The evidence included EV-associated ncRNAs, retroelement-associated cargo, and changes in macrophage EV composition or biological activity.

Several studies demonstrated environmentally or disease-responsive alterations in EV cargo and effects on recipient cells (Feng et al., 2025; Otsuki et al., 2021; Pantazi et al., 2022; Zhong et al., 2024). These findings provide Mechanistic evidence that EVs can communicate molecular states between cells and intersect directly with the ncRNA, transposable-element, and signaling modules. Other studies provided Contextual evidence for retroelement-associated or environmentally responsive EV cargo (Khatua et al., 2010; Saba et al., 2025).

The central distinction revealed by the audit is between information transfer and memory transfer. Demonstrating that an EV carries RNA, protein, retroelement-derived material, or another regulatory molecule and changes the phenotype of a recipient cell establishes intercellular signaling, but it does not demonstrate environmental memory. Direct evidence would additionally require showing that the transferred information establishes a recipient-cell state that persists after the EV signal is removed, is maintained over time or through cell division, or modifies a subsequent response.

Tassetto et al., in a seminal paper, provided the single Direct study assigned to this module. In a *Drosophila* antiviral model, macrophage-like circulating immune cells generated persistent antiviral small-RNA information and transferred antiviral small RNAs through extracellular vesicle/exosome-like particles (Tassetto et al., 2017). This study is particularly relevant because it connects persistence of environmentally acquired molecular information with its intercellular communication rather than demonstrating EV transfer alone.

Thus, EV-mediated communication represents a plausible mechanism for propagating environmentally induced information, but direct evidence that EVs transfer persistent macrophage environmental memory remains extremely limited. The critical next experiment is to transfer defined, traceable EV populations from environmentally challenged macrophages to naïve recipient macrophages, remove the EV exposure, and longitudinally determine whether molecular or functional alterations persist and modify responses to subsequent challenge. Such experiments would directly test whether EVs communicate transient signals or durable cellular memory.

### 3.6 Metabolic and signaling reprogramming

Metabolic and signaling reprogramming was represented in 86 studies, including 4 Direct, 36 Mechanistic, and 46 Contextual studies, making it the second-largest MEMS module. This broad category encompassed inflammatory and interferon signaling, innate nucleic-acid sensing, macrophage differentiation and polarization pathways, metabolic regulation, and interactions among signaling, chromatin, ncRNAs, and transposable elements. The large evidence base demonstrates extensive environmental responsiveness of macrophage signaling networks, but the audit emphasizes that activation or polarization during an ongoing stimulus is not, by itself, evidence of memory.

A substantial portion of this module involved signaling initiated by retroelement- or virus-associated nucleic acids. Early and subsequent studies demonstrated relationships among endogenous or exogenous retroviral material, macrophage or monocyte responses, and inflammatory or antiviral signaling (Bermudez et al., 2015; Herzner et al., 2021; Dopkins et al., 2023; Setoguchi et al., 1989; Leslie et al., 1991; McIntyre et al., 2003; Kumar et al., 2004; Al-Shehabi and Bannert, 2023; Dang and Levin, 2000; Pogue-Geile et al., 1998; Pogue-Geile and Greenberger, 2000; Nellaker et al., 2006; Antony et al., 2007; Buckner et al., 2011; Soe et al., 2011; White et al., 2014; Choi et al., 2015; van Horssen et al., 2016; Morandi et al., 2018; Macchietto et al., 2020; Liu et al., 2024; Sharma et al., 2026). Collectively, these studies support a mechanistic bridge between environmentally responsive nucleic acids and inflammatory or interferon signaling, but most did not test persistence after the initiating stimulus was removed.

A second major group examined pathways associated with macrophage differentiation, polarization, activation, and disease-associated phenotypes (Jiang et al., 2026; Al-Shehabi and Bannert, 2023; Takayama et al., 1991; Blumenstein et al., 1998; Wang et al., 1998; Krichevsky et al., 1999; Johnston et al., 2001; Rolland et al., 2006; Kremer et al., 2019; Matsuzawa et al., 2021; Mikhalkevich et al., 2021; Garcia-S et al., 2024; Bahrami et al., 2018). These studies demonstrate that macrophage regulatory networks can be extensively remodeled by their cellular and tissue environments. Under the audit criteria, however, polarization, differentiation, or disease-associated activation was classified as Mechanistic or Contextual unless the altered state was shown to persist independently of the initiating environment.

Metabolic regulation was less frequently isolated as a primary focus within the mapped evidence base but provided an important mechanistic bridge between environmental sensing and other MEMS modules (Simoes-Barbosa et al., 2006). Metabolic state can influence inflammatory signaling and potentially chromatin-regulatory processes, while signaling pathways reciprocally alter cellular metabolism. The evidence therefore supports close coupling of metabolism and signaling within the framework, but prospective studies are required to determine when such changes become self-maintaining rather than reflecting ongoing environmental conditions.

The four Direct studies in the complete evidence set all intersected with metabolic or signaling regulation according to the final module audit. Wu et al. demonstrated maintenance of a piRNA-induced regulatory state after removal of the initiating signal and through cell division (Wu et al., 2015). Tassetto et al. demonstrated continued antiviral small-RNA production after disappearance of the initiating viral RNA together with EV-mediated communication (Tassetto et al., 2017). The two post-acute COVID studies demonstrated persistent HERV-associated and epigenetic/myeloid states following the acute infectious period (Koo and Morrow, 2025; Koo and Morrow, 2026). These studies differ from conventional activation studies because persistence, rather than pathway activation alone, is central to their classification as Direct evidence.

The major knowledge gap in this module is therefore temporal and causal. Signaling activation, macrophage polarization, metabolic adaptation, and inflammatory phenotype are common responses to environmental stress, but they should not be equated with memory while the initiating stimulus or disease microenvironment remains present. Experiments incorporating stimulus withdrawal, longitudinal metabolic and signaling measurements, targeted pathway perturbation, and functional rechallenge will be necessary to determine which signaling states initiate memory, which maintain it, and which merely accompany an ongoing environmental response.

### 3.7 Systems integration

Systems integration was represented in 17 studies, comprising 2 Direct, 7 Mechanistic, and 8 Contextual studies. This module captured evidence that could not be adequately represented by a single molecular mechanism and instead linked multiple MEMS domains through single-cell, genomic, epigenomic, transcriptomic, or other systems-level approaches. The relatively small number of studies reflects the emerging nature of integrative analyses rather than evidence that cross-module interactions are uncommon.

Single-cell and multi-omic approaches provided much of the Mechanistic and Contextual evidence for systems integration. These studies identified coordinated changes across transcriptional, epigenetic, retroelement, signaling, and cell-state programs in macrophage or monocyte populations exposed to disease-associated or environmentally altered microenvironments (Gerlevik et al., 2024; Du et al., 2024; Allgood et al., 2025; Al-Shehabi and Bannert, 2023; Sakashita et al., 2020; Arteaga-Vazquez et al., 2025; Cooles et al., 2025; Funaguma et al., 2025). Such approaches are particularly valuable for MEMS because they can identify relationships among molecular modules within the same cells rather than treating epigenetic regulation, transposable elements, metabolism, and inflammatory signaling as independent processes.

Systems/genomic analyses in disease-associated immune states provided additional Contextual evidence for coordinated molecular regulation (Broce et al., 2018). Cross-module regulatory analyses likewise demonstrated relationships among molecular systems relevant to macrophage state and environmental responsiveness (Jiang et al., 2017). Together, these studies support the biological rationale for organizing the literature as an interacting systems framework. However, cross-sectional multi-omic correlations do not establish environmental memory, even when multiple MEMS modules are simultaneously altered.

The Direct evidence within the systems-integration module was limited to two studies in the final audit. In particular, the persistent post-acute molecular states identified in the Koo studies provide a systems-level example in which retroelement activity, epigenetic regulation, and immune-cell state remain altered beyond the acute initiating condition (Koo and Morrow, 2025; Koo and Morrow, 2026). Their importance for MEMS lies not simply in the number of molecular pathways altered, but in the temporal persistence of an integrated state. By contrast, systems-level differences measured only during active disease or continuous environmental stimulation remained Mechanistic or Contextual.

The systems-level synthesis also emphasizes that the six MEMS modules are interconnected and non-mutually exclusive rather than six obligatory steps in a single pathway. Epigenetic regulation can alter transposable-element activity; transposable-element-derived nucleic acids can activate inflammatory signaling; ncRNAs can regulate both chromatin and transposable elements; metabolic state can influence chromatin-regulatory enzymes and signaling networks; and EVs can communicate regulatory molecules among cells. The evidence supports studying these interactions as an integrated regulatory architecture, but it does not demonstrate that all six modules are required for environmental memory in any individual macrophage.

Accordingly, the principal need in systems-level studies is to add time and causality to multi-omic integration. Prospective experimental designs should follow defined macrophage or monocyte populations through environmental challenge, stimulus withdrawal, recovery, and rechallenge while simultaneously measuring multiple MEMS modules. Lineage tracing, single-cell and spatial multi-omics, targeted genetic or epigenetic perturbation, and longitudinal functional assays could then distinguish molecular changes that merely correlate with an environmental response from regulatory networks that causally maintain a persistent state. Such studies would provide the strongest test of the MEMS Framework as a systems-level model of macrophage environmental memory.

## 4 Discussion

This PRISMA-guided systematic review identified 119 primary studies relevant to macrophage environmental memory, including only 4 Direct studies compared with 50 Mechanistic and 65 Contextual studies. This marked imbalance indicates that environmentally responsive macrophage biology is extensively documented, whereas direct evidence of persistent environmental memory remains limited.

The small Direct evidence set is particularly informative. In U937 cells, a CDKN2B-associated piRNA induced DNA and histone methylation changes that were reported to persist after removal of the initiating piRNA and through subsequent cell divisions (Wu et al., 2015). In *Drosophila* macrophage-like circulating immune cells, reverse-transcribed viral information supported continued production of antiviral small RNAs after the initiating viral RNA was no longer detectable, together with EV-mediated transfer of antiviral small RNAs (Tassetto et al., 2017). Two recent studies further identified persistent HERV-associated transcriptional states in post-acute COVID-19 monocyte or related mononuclear-cell populations (Koo and Morrow, 2025; Koo and Morrow, 2026). Although these studies differ substantially in biological model and mechanism, they share the critical temporal feature distinguishing environmental memory from an ongoing environmental response.

The much larger Mechanistic and Contextual evidence base identifies numerous candidate mechanisms that could potentially support such persistent states. Epigenetic regulation, ncRNAs, TEs, innate immune signaling, metabolism, and EV communication can interact extensively in macrophages and related cells (Wu et al., 2015; Bermudez et al., 2015; Tassetto et al., 2017; Herzner et al., 2021; Zhong et al., 2024; Du et al., 2025). These interactions provide a biological rationale for a systems-level framework, but they should not be interpreted as proof that environmental memory is necessarily an emergent property of their combined activity. Instead, they identify specific interactions that can now be tested prospectively using exposure-withdrawal, longitudinal, perturbational, and rechallenge designs.

### 4.1 MEMS as an evidence-organizing systems framework

A principal contribution of MEMS is to place molecular mechanisms that have generally been studied separately into a common conceptual structure. Epigenetic regulation can influence transcription and TE activity; ncRNAs can regulate gene expression, chromatin, and signaling; TE-derived nucleic acids can interact with innate immune pathways; metabolic and inflammatory signaling can alter transcriptional regulation; and EVs can transfer regulatory molecules between cells (Neidhart et al., 2000; Wu et al., 2015; Bermudez et al., 2015; Tassetto et al., 2017; Herzner et al., 2021; Zhong et al., 2024; Du et al., 2025). The evidence therefore supports investigating these mechanisms as interacting regulatory systems rather than if each operates independently.

The strength of evidence, however, varies markedly among MEMS modules. TE regulation was represented by 107 studies, followed by metabolic and signaling reprogramming (86), epigenetic regulation (33), systems integration (17), ncRNA regulation (14), and EV-mediated communication (7). These counts are non-mutually exclusive because individual studies could contribute to multiple modules. More importantly, the number of studies assigned to a module should not be equated with evidence that the module generates environmental memory. This distinction is evident in Table 1, where the module-level evidence is separated into Direct, Mechanistic, and Contextual categories.

Epigenetic regulation provides a useful example. The module contained 33 studies, but only 2 were classified as Direct, compared with 13 Mechanistic and 18 Contextual studies. Environmentally responsive DNA methylation, histone modification, and chromatin remodeling are therefore well represented in the literature, but most studies did not test persistence after stimulus withdrawal or altered subsequent responsiveness (Neidhart et al., 2000; Bouttier et al., 2016; Li et al., 2020; Du et al., 2025). The distinction between epigenetic response and epigenetic memory is illustrated in Figure 3, in which persistence or recall functions as the evidentiary gate separating environmentally induced epigenetic change from Direct evidence of memory.

The same caution applies to ncRNAs, TEs, and EVs. These systems are biologically interconnected: ncRNAs can regulate chromatin and TE activity, TE-derived nucleic acids can engage innate immune signaling, and EVs can transport RNAs and other regulatory molecules between cells (Wu et al., 2015; Feng et al., 2025; Bermudez et al., 2015; Tassetto et al., 2017; Pantazi et al., 2022; Zhong et al., 2024). These candidate cross-module relationships are summarized in Figure 4. However, the evidence profiles remain predominantly Mechanistic or Contextual: ncRNA regulation comprised 1 Direct, 9 Mechanistic, and 4 Contextual studies; TE regulation, 3 Direct, 41 Mechanistic, and 63 Contextual; and EV-mediated communication, 1 Direct, 4 Mechanistic, and 2 Contextual. Thus, Figure 4 should be interpreted as a map of candidate interactions rather than a demonstrated pathway producing environmental memory.

**FIGURE 4:**
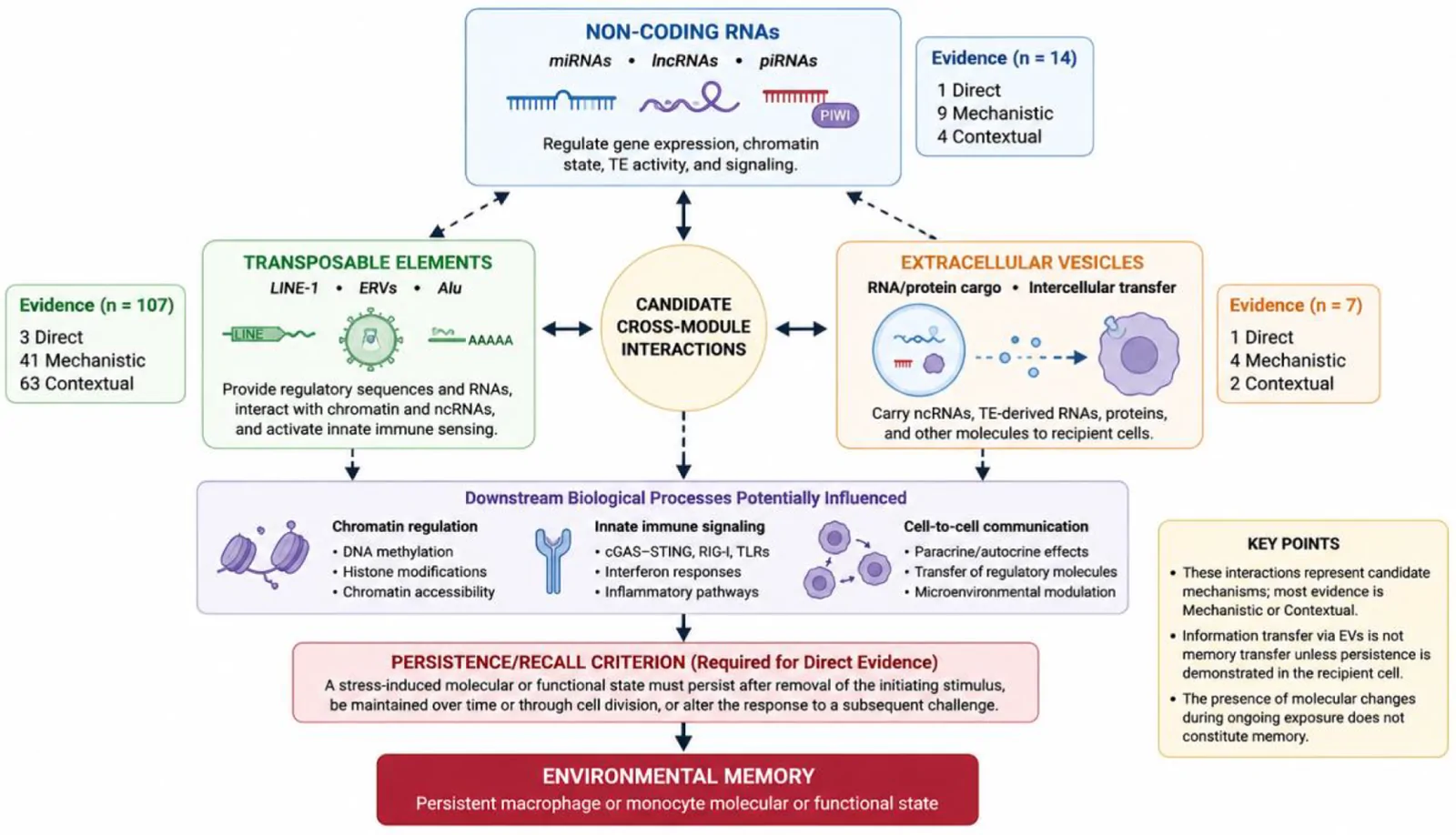
Candidate interactions among non-coding RNAs, transposable elements, and extracellular vesicle-mediated communication relevant to macrophage environmental memory. Non-coding RNAs (ncRNAs), transposable elements (TEs), and extracellular vesicles (EVs) represent interconnected MEMS modules that may influence chromatin regulation, innate immune signaling, and cell-to-cell communication. ncRNAs can regulate gene expression, chromatin state, TE activity, and signaling, whereas TE-derived nucleic acids can interact with epigenetic and innate immune pathways. EVs can transfer ncRNAs, TE-derived RNAs, proteins, and other regulatory molecules between cells. The evidence base comprised 14 ncRNA studies (1 Direct, 9 Mechanistic, 4 Contextual), 107 TE studies (3 Direct, 41 Mechanistic, 63 Contextual), and 7 EV studies (1 Direct, 4 Mechanistic, 2 Contextual); module assignments were non-mutually exclusive. These interactions represent candidate mechanisms rather than direct evidence of environmental memory. Under the operational definition used in this review, Direct evidence requires that an induced molecular or functional state persist after removal of the initiating stimulus, be maintained over time or through cell division, or alter the response to a subsequent challenge. Importantly, EV-mediated transfer of molecular information does not constitute transfer of memory unless a persistent state is demonstrated in the recipient cell. Abbreviations: circRNA, circular RNA; ERV, endogenous retrovirus; EV, extracellular vesicle; lncRNA, long non-coding RNA; MEMS, Macrophage Environmental Memory Systems; miRNA, microRNA; ncRNA, non-coding RNA; piRNA, PIWI-interacting RNA; PIWI, P-element-induced wimpy testis protein; TE, transposable element; TLR, Toll-like receptor.

This distinction is particularly important for EV biology. Macrophage EVs can carry regulatory RNAs, proteins, lipids, and other molecules capable of affecting recipient cells (Feng et al., 2025; Otsuki et al., 2021; Pantazi et al., 2022; Zhong et al., 2024). Tassetto et al. provide Direct evidence of persistence of antiviral molecular information together with EV-mediated transfer in *Drosophila* macrophage-like cells (Tassetto et al., 2017). Nevertheless, information transfer is not equivalent to memory transfer. Demonstrating that EV cargo modifies a recipient cell does not establish transfer of environmental memory unless the induced recipient-cell state itself persists after the transferred signal is removed or modifies a subsequent response.

Systems integration similarly represents an organizational level of the MEMS Framework rather than a molecular mechanism demonstrated in isolation. Studies assigned to this module provided data spanning multiple molecular layers, cell populations, or regulatory mechanisms (Koo and Morrow, 2025; Koo and Morrow, 2026; Jiang et al., 2017; Gerlevik et al., 2024; Du et al., 2024). Only 2 of the 17 systems-integration studies met the Direct criterion. Thus, multi-omic association alone should not be interpreted as evidence of an integrated memory state. The major experimental opportunity is to add temporal and causal dimensions to systems-level studies by following defined macrophage or monocyte populations through exposure, withdrawal, recovery, and rechallenge.

### 4.2 Relationship between MEMS and immunity

Trained immunity provides an important conceptual foundation for macrophage environmental memory (reviewed in (Bhargavi and Subbian, 2024)). It established that innate immune cells could exhibit altered responses following a previous challenge and identified epigenetic and metabolic reprogramming as candidate mechanisms underlying such persistence (Daskalaki et al., 2025). MEMS is therefore not intended to replace trained immunity. Rather, it provides a broader organizational framework for evaluating molecular mechanisms relevant to persistent macrophage states across a wider range of environmental and tissue contexts.

The distinction is partly one of scope and partly one of evidentiary emphasis. Trained-immunity experiments frequently use a defined primary stimulus, a recovery interval, and a secondary challenge, providing an experimental structure well suited to testing memory. MEMS encompasses these responses but also considers mechanisms identified during infection, inflammatory and autoimmune disease, metabolic perturbation, cancer and the tumor microenvironment, tissue injury, and environmental chemical exposure. This broader scope makes the strict persistence criterion particularly important because molecular differences observed during an ongoing disease or exposure cannot automatically be interpreted as memory (reviewed in (Li et al., 2024)).

MEMS also incorporates regulatory systems that are not usually central to classical descriptions of trained immunity, particularly ncRNAs, TEs, EV-mediated communication, and systems-level interactions. Experimental studies support biological connections among these processes and more established chromatin and signaling pathways (Wu et al., 2015; Bermudez et al., 2015; Tassetto et al., 2017; Herzner et al., 2021; Zhong et al., 2024). The present systematic review therefore suggests that these mechanisms warrant investigation in experimental paradigms like those that established trained immunity: defined exposure, stimulus withdrawal, recovery, and functional or molecular rechallenge.

An important implication is that trained immunity should not be treated as synonymous with all macrophage environmental memory. Conversely, the presence of a molecular mechanism incorporated into MEMS does not establish trained immunity or environmental memory. MEMS instead provides a framework for asking which environmentally responsive mechanisms satisfy the persistence or recall criteria summarized in Box 1.

### 4.3 Biological and translational implications

The MEMS Framework generates several potential translational applications, but these should be considered hypotheses arising from the evidence synthesis rather than established clinical applications. Persistent macrophage molecular states could potentially provide biomarkers of prior environmental or tissue conditions, particularly if such states remain detectable after the initiating exposure has ended. Epigenetic signatures, ncRNAs, TE or ERV expression, EV cargo, and metabolic or signaling profiles therefore represent candidate biomarker classes (Neidhart et al., 2000; Wu et al., 2015; Koo and Morrow, 2025; Koo and Morrow, 2026).

The Direct studies illustrate the type of temporal information that would strengthen biomarker development. Persistent HERV-associated transcriptional changes after acute COVID-19, for example, suggest that molecular states measured after an initiating disease process may retain information about prior biological conditions (Koo and Morrow, 2025; Koo and Morrow, 2026). Whether such signatures are sufficiently stable, specific, reproducible, and predictive for clinical or exposure assessment remains to be established.

The framework also identifies candidate intervention points. Epigenetic regulators, ncRNA pathways, TE-associated innate immune signaling, EV communication, and metabolic or inflammatory signaling are potentially modifiable processes (Bermudez et al., 2015; Herzner et al., 2021; Pantazi et al., 2022; Zhong et al., 2024; Du et al., 2025). However, therapeutic manipulation of an environmentally responsive pathway should not be interpreted as targeting “memory” unless that pathway is first shown to causally maintain a persistent state. Establishing causality will require perturbation experiments in which candidate mechanisms are disrupted during memory acquisition, maintenance, or recall.

Systems-level approaches may ultimately be particularly useful because persistent states could involve combinations of molecular alterations rather than individual biomarkers (reviewed in (Karkossa et al., 2020)). Single-cell transcriptomics, epigenomics, chromatin accessibility profiling, EV characterization, metabolomics, spatial methods, and computational network analysis could be combined to identify persistent molecular states across multiple MEMS modules (Gerlevik et al., 2024; Du et al., 2024; Allgood et al., 2025). Their greatest value for MEMS will come from combining these technologies with longitudinal experimental design rather than relying on cross-sectional multi-omic differences alone.

### 4.4 Limitations of the evidence base and the MEMS framework

The most important limitation of the evidence base is also one of the principal findings of this review: only 4 of 119 included primary studies met the Direct criterion. Most of the literature therefore establishes environmental responsiveness or mechanistic plausibility rather than persistent environmental memory. This imbalance requires caution in interpreting both the individual MEMS modules and the framework.

The four Direct studies are themselves heterogeneous. They include a human U937 cell-line model, *Drosophila* macrophage-like circulating immune cells, and human post-acute disease-associated monocyte or mononuclear-cell states (Wu et al., 2015; Tassetto et al., 2017; Koo and Morrow, 2025; Koo and Morrow, 2026). Consequently, they should not be interpreted as four independent demonstrations of a single conserved macrophage-memory mechanism. Instead, they establish that persistent environmentally induced molecular states can occur in macrophage-related systems and identify candidate mechanisms requiring replication and extension.

The evidence is also highly heterogeneous with respect to experimental model, stressor, disease context, macrophage or monocyte population, exposure duration, follow-up period, and molecular endpoint. This heterogeneity precluded meaningful quantitative meta-analysis and limits direct comparison among studies. In addition, most studies were not originally designed to test environmental memory, explaining why the Mechanistic and Contextual categories substantially outnumber Direct studies.

The evidence profiles also differ substantially among modules. For example, TE regulation was represented in 107 studies, whereas only 7 studies were assigned to EV-mediated communication. Conversely, even the four studies assigned as Direct within the combined metabolic-and-signaling module primarily demonstrated persistent epigenetic/piRNA, antiviral small-RNA, or HERV-associated transcriptional states rather than persistent metabolic reprogramming itself (Wu et al., 2015; Tassetto et al., 2017; Koo and Morrow, 2025; Koo and Morrow, 2026). Direct evidence specifically demonstrating persistent metabolic reprogramming therefore remains limited.

Several methodological limitations of the systematic review should also be acknowledged. Study identification, screening, extraction, annotation, and final evidence classification were conducted by a single author rather than by independent duplicate reviewers. To increase transparency, all 132 candidate records underwent an additional final manual audit, the 13 records excluded during this audit were retained with reasons for exclusion in Supplementary Table S2, and the complete final 119-study evidence atlas and study-level classifications are provided in the supplementary material. The diversity of study designs also prevented application of a single risk-of-bias instrument across the complete evidence set. Accordingly, the Direct, Mechanistic, and Contextual categories describe relationship to the environmental-memory question rather than methodological quality. These limitations are consistent with those identified in the revised Methods.

Finally, MEMS was developed through synthesis of the literature rather than prospectively specified and experimentally tested. Systems integration is therefore partly an organizational construct of this review. The available literature supports biological interactions among MEMS modules but does not demonstrate that the six-module architecture itself constitutes a biological memory pathway. MEMS should consequently remain explicitly framed as an evidence-organizing framework whose proposed interactions require prospective validation.

### 4.5 Future research priorities and experimental testing of MEMS

The most immediate research priority is to convert the large Mechanistic evidence base into experiments that directly test persistence. A useful minimum experimental design would include a defined environmental challenge, characterization of the initial macrophage response, removal of the initiating stimulus, an appropriate recovery period, and reassessment of the molecular and functional state. Rechallenge should be incorporated when biologically appropriate to determine whether the prior exposure modifies subsequent responsiveness. These designs would directly distinguish transient environmental responses from persistent memory.

Causal perturbation should accompany temporal studies. Candidate epigenetic regulators, ncRNAs, TEs, innate immune sensors, metabolic pathways, and EV-mediated signals can be experimentally disrupted during the acquisition, maintenance, or recall phases of a putative memory state. Such studies could determine whether a candidate mechanism merely correlates with persistence or is required to establish or maintain it. The cross-module relationships summarized in Figures 3, 4 provide specific hypotheses for such experiments.

Systems-level testing will require simultaneous measurement of multiple MEMS modules across time. Longitudinal single-cell and spatial multi-omics, lineage tracing, chromatin and epigenetic profiling, TE-specific transcriptomics, small-RNA sequencing, EV profiling, metabolomics, and functional assays could be integrated across exposure, withdrawal, recovery, and rechallenge (Gerlevik et al., 2024; Du et al., 2024; Allgood et al., 2025). Such designs could determine whether persistent states involve coordinated changes across modules and whether those relationships are causal or simply correlated consequences of environmental exposure.

Systems-level testing of MEMS may also benefit from computational approaches that integrate single-cell, genomic, transcriptomic, and other multi-omic data with machine learning. Recent cancer studies illustrate how such approaches can identify cell-associated molecular signatures, integrate heterogeneous molecular datasets, and relate them to complex biological phenotypes. Although these studies do not demonstrate macrophage environmental memory, they provide methodological examples for future longitudinal MEMS studies in which multi-omic measurements are integrated across exposure, withdrawal, recovery, and rechallenge (Zhu et al., 2025; Zhu et al., 2023a; Zhu et al., 2023b).

Future studies should also distinguish cell-autonomous memory from intercellular information transfer. This distinction is particularly important for EVs. Experiments should determine whether EVs produced by environmentally challenged macrophages induce a durable state in naïve recipient cells and whether that state remains after the transferred EVs or their initiating cargo are no longer present. Tassetto et al. provide an important foundation for this question but do not establish that EV transfer itself generates persistent memory in recipient cells (Tassetto et al., 2017).

Finally, the Macrophage-Mediated Molecular Memory (M3) hypothesis represents one downstream testable extension of MEMS rather than a conclusion of the systematic review (Ruden, 2026). The possibility that macrophage-derived EVs containing piRNAs or other regulatory molecules could communicate environmentally acquired information to distant tissues or germ cells requires direct experimental demonstration of transfer, uptake, molecular function, persistence, and ultimately phenotypic consequences in recipient cells or subsequent generations (Ruden, 2026). These requirements provide a clear experimental path for evaluating M3 without making the validity of the broader MEMS Framework dependent on intergenerational inheritance.

Taken together, the revised evidence base shifts the central question from whether macrophages possess numerous environmentally responsive molecular mechanisms—which is well supported—to which of these mechanisms establish, maintain, or communicate persistent environmental memory. Answering that question will require moving beyond cross-sectional molecular associations toward temporal, causal, and rechallenge-based experimental designs. In this context, the primary value of MEMS is not that it establishes a new biological pathway, but that it provides a structured framework for identifying and experimentally testing the molecular systems most likely to contribute to persistent macrophage environmental states.

## 5 Conceptual framework and perspective

The Macrophage Environmental Memory Systems (MEMS) Framework provides an evidence-organizing systems perspective for investigating how macrophages and monocytes may acquire, maintain, integrate, and communicate molecular information generated by prior environmental or tissue-derived stressors. The framework encompasses six interconnected and non-mutually exclusive molecular systems: epigenetic regulation, non-coding RNA regulation, transposable element regulation, extracellular vesicle-mediated communication, metabolic and signaling reprogramming, and systems integration (Figure 1; Table 1). These modules emerged from synthesis of the literature but should not be interpreted as six obligatory components of a single biological pathway.

A central conceptual contribution of MEMS is the explicit separation of environmental response from environmental memory (Box 1). Macrophages undergo extensive molecular remodeling during infection, inflammation, metabolic perturbation, cancer, tissue injury, and other changes in their microenvironment (Neidhart et al., 2000; Bermudez et al., 2015; Herzner et al., 2021; Zhong et al., 2024; Du et al., 2025). Such environmentally responsive states may identify mechanisms capable of contributing to memory, but they do not constitute memory unless persistence or altered subsequent responsiveness is demonstrated. This distinction is reinforced by the final evidence audit: among 119 primary studies, only 4 met the Direct criterion, compared with 50 Mechanistic and 65 Contextual studies.

The Direct studies illustrate several ways in which persistence can potentially be encoded. A piRNA-induced DNA and histone methylation state persisted after removal of the initiating piRNA and through subsequent cell divisions in U937 cells (Wu et al., 2015). *Drosophila* macrophage-like immune cells retained reverse-transcribed viral information capable of supporting continued antiviral small-RNA production after the initiating viral RNA was no longer detectable (Tassetto et al., 2017). Persistent HERV-associated transcriptional states have also been identified in monocyte or related mononuclear-cell populations following acute COVID-19 (Koo and Morrow, 2025; Koo and Morrow, 2026). These heterogeneous examples do not establish a common macrophage-memory pathway, but they demonstrate the types of temporal evidence needed to distinguish persistent molecular information from transient environmental responses.

The broader Mechanistic and Contextual literature suggests numerous potential interactions among MEMS modules. Epigenetic regulation can interact with ncRNAs and TEs; retroelement-derived molecules can engage innate immune signaling; metabolic and inflammatory pathways can modify transcriptional regulation; and EVs can transfer regulatory molecules between cells (Wu et al., 2015; Bermudez et al., 2015; Tassetto et al., 2017; Herzner et al., 2021; Zhong et al., 2024; Du et al., 2025). Figures 3, 4 illustrate two sets of candidate relationships particularly suitable for experimental testing: the transition from environmentally responsive epigenetic remodeling to persistent epigenetic memory, and interactions among ncRNAs, TEs, and EV-mediated communication. These figures represent testable mechanisms rather than established pathways of environmental memory.

This distinction also clarifies the relationship between MEMS and the previously proposed Macrophage-Mediated Molecular Memory (M3) hypothesis (Ruden, 2026). MEMS addresses the broader question of how environmentally induced information may be acquired, maintained, and communicated by macrophages. M3 represents a more specific downstream hypothesis proposing that macrophage-derived EVs could transport environmentally responsive regulatory molecules, potentially including piRNAs, to other cells and, under appropriate biological conditions, potentially to germ cells (Ruden, 2026). The evidence reviewed here supports biological components relevant to this hypothesis—including small-RNA regulation, EV communication, and persistent molecular states—but does not demonstrate macrophage-to-germline transfer or intergenerational inheritance. M3 therefore remains a testable hypothesis rather than a conclusion of the systematic review.

This hierarchy is important conceptually. MEMS does not depend on M3 being correct, and evidence supporting individual MEMS modules does not validate the complete MEMS Framework. Instead, the framework provides a structured means of converting a heterogeneous literature into experimentally testable questions. Which environmentally induced macrophage states persist after stimulus withdrawal? Which molecular mechanisms are required for their maintenance? Can molecular information be transferred between cells, and does the recipient state itself persist? Do multiple MEMS modules cooperate causally or merely change in parallel? These questions provide direct routes for testing and, where necessary, revising the framework.

Accordingly, the principal value of MEMS is its falsifiability and experimental utility rather than its status as an established biological model. Prospective exposure-withdrawal, longitudinal, perturbational, lineage-tracing, and rechallenge experiments can determine which proposed mechanisms genuinely contribute to macrophage environmental memory. Multi-omic studies can further test whether persistent states involve coordinated changes across MEMS modules. The framework should therefore evolve as Direct evidence accumulates, with unsupported relationships modified or discarded rather than incorporated as assumptions.

## 6 Conclusion

This PRISMA-guided systematic review identifies a substantial literature describing molecular mechanisms potentially relevant to macrophage environmental memory but a much smaller body of evidence directly demonstrating persistence. Of 119 included primary studies, only 4 met the stringent criteria for Direct evidence, whereas 50 were classified as Mechanistic and 65 as Contextual. This imbalance is a major finding: environmentally responsive macrophage biology is extensively documented, but environmental response should not be equated with environmental memory.

The most important next step is experimental. Future studies should incorporate defined exposure and withdrawal periods, longitudinal assessment, causal perturbation, and, where appropriate, rechallenge to determine whether environmentally induced macrophage states persist and which mechanisms are required for their maintenance. Attention should be given to distinguishing epigenetic change from epigenetic memory, information transfer from memory transfer, and multi-omic association from causal systems integration.

Within this framework, the M3 hypothesis represents one downstream and experimentally testable possibility involving the communication of environmentally acquired molecular information from macrophages to other cells (Ruden, 2026). Its proposed extension to germ cells and intergenerational inheritance remains speculative and requires direct experimental validation. MEMS can therefore be tested independently of M3.

Overall, the evidence supports a shift in emphasis from asking whether macrophages undergo molecular responses to environmental stress—which is well established—to determining which responses persist, how they are maintained, whether they modify subsequent responses, and whether persistent information can be communicated between cells. Addressing these questions should establish whether the candidate mechanisms organized by MEMS constitute genuine components of macrophage environmental memory and define their relevance to human health and disease.

## Data Availability

The original contributions presented in the study are included in the article/Supplementary Material, further inquiries can be directed to the corresponding author.
