# Supplementary material for: Environmental memory in macrophages: a PRISMA-guided systematic review and the macrophage environmental memory systems framework
Source: Front Syst Biol. 2026 Oct 21;6:1955135. doi: 10.3389/fsysb.2026.1955135 (PMC13730966; doi:10.3389/fsysb.2026.1955135)
Supplement: Supplementary file 1 [file DataSheet1.pdf]

Supplementary Figure S1. PRISMA 2020 Checklist

Environmental Memory in Macrophages: A PRISMA-Guided Systematic Review and the Macrophage Environmental Memory Systems (MEMS) Framework

Completed for the revised manuscript. “N/A” indicates an item not applicable to this qualitative, heterogeneous evidence synthesis.

Section and topic	Item	PRISMA 2020 checklist item	Location in revised manuscript
TITLE	1	Identify the report as a systematic review.	Title
ABSTRACT	2	See the PRISMA 2020 for Abstracts checklist.	Abstract
INTRODUCTION - Rationale	3	Describe the rationale for the review in the context of existing knowledge.	Introduction, Sections 1.1-1.4
INTRODUCTION - Objectives	4	Provide an explicit statement of the objective(s) or question(s) the review addresses.	Introduction, end of Section 1.4
METHODS - Eligibility criteria	5	Specify inclusion and exclusion criteria and how studies were grouped for synthesis.	Sections 2.2-2.5; Supplementary Tables S1-S2
METHODS - Information sources	6	Specify all databases, registers, websites, organizations, reference lists and other sources searched or consulted, and the date each source was last searched.	Section 2.1; Supplementary Table S1 (PubMed and Web of Science; final search August 1, 2026)
METHODS - Search strategy	7	Present the full search strategies for all databases, registers and websites, including filters and limits used.	Section 2.1; Supplementary Table S1
METHODS - Selection process	8	Specify methods used to decide whether a study met inclusion criteria, including number of reviewers and any automation used.	Sections 2.2 and 2.7; single-author screening and final manual audit
METHODS - Data collection process	9	Specify methods used to collect data from reports, including number of reviewers and any automation used.	Sections 2.3-2.5 and 2.7; Supplementary Table S2
METHODS - Data items: outcomes	10a	List and define all outcomes for which data were sought and describe assumptions made about missing or unclear information.	Sections 2.3-2.5; operational Direct/Mechanistic/Contextual criteria
METHODS - Data items: other variables	10b	List and define other variables for which data were sought and describe assumptions made about missing or unclear information.	Sections 2.3-2.5; Supplementary Table S2
METHODS - Study risk of bias	11	Specify methods used to assess risk of bias in included studies.	Section 2.7; no uniform formal risk-of-bias instrument applied because of heterogeneous study designs; stated limitation
METHODS - Effect measures	12	Specify effect measure(s) used for each outcome.	N/A - qualitative mechanistic synthesis; no pooled intervention-effect outcomes
METHODS - Synthesis methods	13a	Describe processes used to decide which studies were eligible for each synthesis.	Sections 2.3-2.6
METHODS - Synthesis methods	13b	Describe methods required to prepare data for presentation or synthesis.	Sections 2.3-2.6; Supplementary Tables S2-S4
METHODS - Synthesis methods	13c	Describe methods used to tabulate or visually display results.	Sections 2.5-2.6; Table 1; Figures 1-4; Supplementary Tables S2-S4
METHODS - Synthesis methods	13d	Describe methods used to synthesize results and rationale for chosen methods.	Sections 2.5-2.7; qualitative synthesis because of heterogeneous study designs and endpoints
METHODS - Synthesis methods	13e	Describe methods used to explore possible causes of heterogeneity among study results.	N/A - no statistical heterogeneity analysis; heterogeneity addressed qualitatively in Sections 2.7 and 4.4
METHODS - Synthesis methods	13f	Describe sensitivity analyses conducted to assess robustness of synthesized results.	N/A - no statistical synthesis or sensitivity analysis
METHODS - Reporting bias assessment	14	Describe methods used to assess risk of bias due to missing results in a synthesis.	Not formally assessed; acknowledged as a limitation in Section 2.7/Discussion
METHODS - Certainty assessment	15	Describe methods used to assess certainty/confidence in the body of evidence.	Sections 2.4-2.7; Direct/Mechanistic/Contextual categories describe relationship to the review question, not formal certainty or quality grades
RESULTS - Study selection	16a	Describe results of the search and selection process, ideally using a flow diagram.	Section 2.1; Figure 2: 1,405 identified; 404 duplicates; 1,001 screened; 132 candidates; 13 excluded in final audit; 119 included
RESULTS - Study selection	16b	Cite studies that appeared eligible but were excluded and explain why.	Supplementary Table S2: 13 excluded records with reasons
RESULTS - Study characteristics	17	Cite each included study and present its characteristics.	Results Sections 3.1-3.7; Supplementary Tables S2 and S4
RESULTS - Risk of bias	18	Present risk-of-bias assessments for each included study.	Not performed; explicitly stated in Section 2.7 and Section 4.4
RESULTS - Individual studies	19	For each outcome, present summary statistics and effect estimates where appropriate.	N/A for pooled quantitative outcomes; study-level mechanistic characteristics and classifications are provided in Supplementary Table S2
RESULTS - Syntheses	20a	For each synthesis, briefly summarize characteristics and risk of bias among contributing studies.	Results Sections 3.1-3.7; Table 1; risk-of-bias limitation in Sections 2.7 and 4.4
RESULTS - Syntheses	20b	Present results of all statistical syntheses conducted.	N/A - no meta-analysis or statistical synthesis
RESULTS - Syntheses	20c	Present results of investigations of possible causes of heterogeneity.	N/A - no formal statistical heterogeneity analysis
RESULTS - Syntheses	20d	Present results of sensitivity analyses.	N/A - no sensitivity analyses
RESULTS - Reporting biases	21	Present assessments of risk of bias due to missing results for each synthesis assessed.	Not formally assessed; acknowledged as a limitation
RESULTS - Certainty of evidence	22	Present assessments of certainty/confidence in the body of evidence.	Results Sections 3.1-3.7 and Table 1; Direct/Mechanistic/Contextual categories are explicitly not formal

Section and topic	Item	PRISMA 2020 checklist item	Location in revised manuscript
			certainty grades
DISCUSSION	23a	Provide a general interpretation of results in the context of other evidence.	Discussion, Sections 4 and 4.1-4.5
DISCUSSION	23b	Discuss limitations of the evidence included in the review.	Section 4.4
DISCUSSION	23c	Discuss limitations of the review processes used.	Sections 2.7 and 4.4
DISCUSSION	23d	Discuss implications for practice, policy, and future research.	Sections 4.3-4.5 and Conclusions
OTHER - Registration	24a	Provide registration information, or state that the review was not registered.	Section 2.7; review was not prospectively registered
OTHER - Protocol	24b	Indicate where the review protocol can be accessed, or state that a protocol was not prepared.	Section 2.7; no prospectively registered/deposited protocol
OTHER - Amendments	24c	Describe and explain amendments to information provided at registration or in the protocol.	N/A - no registered protocol
OTHER - Support	25	Describe sources of financial or non-financial support and the role of funders/sponsors.	Funding statement
OTHER - Competing interests	26	Declare any competing interests of review authors.	Conflict of Interest statement
OTHER - Availability	27	Report which data, code, forms, and other materials are publicly available and where they can be found.	Data Availability Statement; Supplementary Tables S1-S4

Source: Page MJ, McKenzie JE, Bossuyt PM, et al. The PRISMA 2020 statement: an updated guideline for reporting systematic reviews. BMJ. 2021;372:n71. The checklist wording is adapted from the PRISMA 2020 checklist (CC BY 4.0).
